# Probing the Phase and Mechanism of Captured CO_2_ in Supercapacitors by Pore Wetting and NMR Spectroscopy

**DOI:** 10.1021/acs.chemmater.6c00749

**Published:** 2026-06-30

**Authors:** Zeke Coady, Malina Seyffertitz, Benjamin J. Rhodes, Thomas Kress, Amelia Turner, Grace Mapstone, Zhen Xu, Oskar Paris, Alexander C. Forse

**Affiliations:** † Yusuf Hamied Department of Chemistry, 2152 University of Cambridge, Lensfield Road, Cambridge CB2 1EW, U.K.; ‡ Department of Materials and Henry Royce Institute, University of Manchester, Oxford Rd, Manchester M13 9PL, U.K.; § Chair of Physics, Montanuniversitaet Leoben, Franz Josef-Straße 18, 8700 Leoben, Austria

## Abstract

Supercapacitive swing adsorption (SSA) is a promising electrochemical CO_2_ capture technique, yet its development is limited by questions surrounding the mechanism of CO_2_ uptake. The phase and speciation of dissolved inorganic carbon in these systems has not been resolved, and open questions remain as to whether CO_2_ is captured as a gaseous or aqueous species, and if aqueous, whether the primary captured species is CO_2_, HCO_3_^–^, or CO_3_^2–^. In this work, we investigate these questions in model SSA systems using solid-state nuclear magnetic resonance (NMR) spectroscopy and small-angle neutron scattering, and identify that the dominant species present are aqueous CO_2_ and HCO_3_^–^. Examination of variably wetted activated carbon/aqueous electrolyte systems dosed with CO_2_ indicates that a previously hypothesized uptake of gaseous CO_2_ in dry pores does not occur in the studied SSA systems. Instead, quantitative NMR spectroscopy measurements reveal a large pool of aqueous CO_2_ and HCO_3_^–^ which adsorbs prior to charging and exceeds the observed uptake of supercapacitive CO_2_ capture. Observation of fast CO_2_:HCO_3_^–^ exchange in this pool indicates that both species would be involved in any SSA process. These findings provide key insights into the mechanism of supercapacitive CO_2_ capture and will guide its future technological development as a method for the mitigation of CO_2_ emissions.

## Graphical abstract

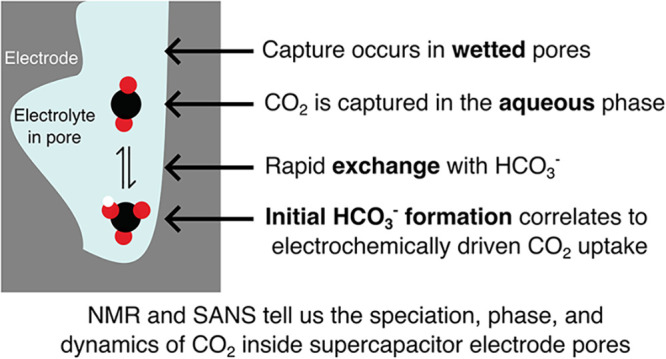

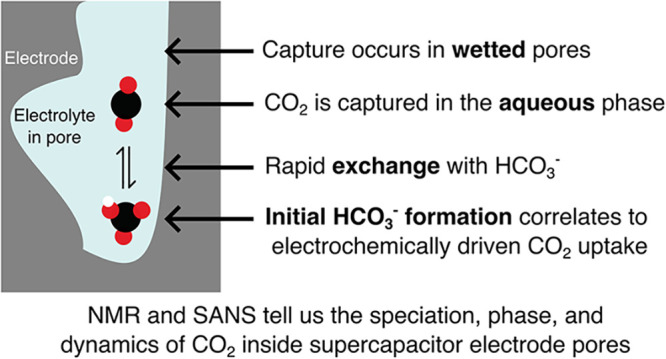

## Introduction

To reach net-zero targets and address the climate crisis, it is critical to develop more effective and efficient CO_2_ capture methods.^1^ Industrial-scale CO_2_ capture is currently performed using amine-based sorbents, relying on a thermal swing to release captured CO_2_, an energetically inefficient and expensive process.^2^ Electrochemical CO_2_ capture methods are promising alternatives due to their independence from Carnot limitations, temperature-independent operation, and ability to be easily powered by renewable energy sources.^3,4^

Supercapacitive swing adsorption (SSA) is a recently developed and particularly promising electrochemical CO_2_ capture method.^5,6^ In an SSA system, CO_2_ is captured upon charging an aqueous electrolyte supercapacitor in contact with the gas phase and released upon discharging, as shown in Figure 1a.

**Figure 1:**
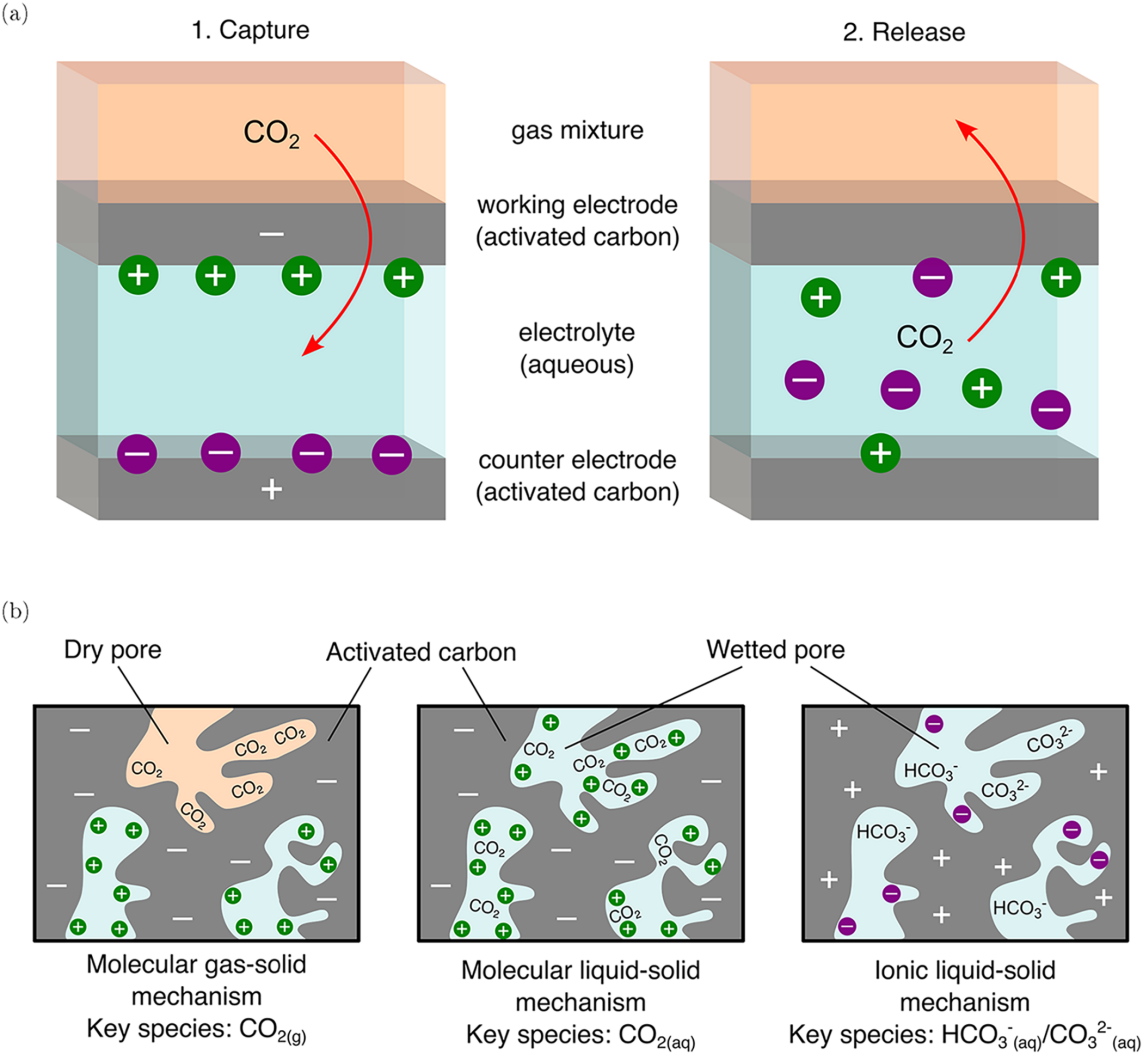
(a) Diagram of SSA capture. Charging results in uptake of CO_2_ across the electrode:gas interface, followed by release upon discharging. (b) Diagram showing proposed mechanisms of action of SSA processes. From left to right: molecular gas–solid, molecular liquid–solid, and ionic liquid–solid.

Studies of SSA demonstrate its stability, high energy efficiency, selectivity for CO_2_ over O_2_, scalability, and its use of low-cost, abundant, and sustainable materials.^7–10^ These qualities set SSA apart from other electrochemical CO_2_ capture methods, which can suffer from low sorbent stability^3,11^ and use more complex components such as redox-active CO_2_-binding small molecules^12,13^ or membranes.^14,15^ However, SSA is limited by low CO_2_ adsorption capacity and rate relative to competing technologies. The maximum reported gravimetric adsorption capacity^9^ of 780 mmol kg^–1^ is comparable to but remains generally lower than adsorption capacities in amine solvents (0.7–2.7 mmol kg^–1^).^16^ Similarly, the adsorption rate as a function of electrode area in SSA has been reported to be 30–110 mmol h^–1^ m^2^ in high-performing setups,^9,17^ which is much lower than the recent report of 82.2 mmol h^–1^ m^2^ (or 0.137 mmol min^–1^ cm^2^) by Zhu et al. (2023)^18^ in an alternate electrochemical CO_2_ capture setup. This is despite significant recent research effort into improving SSA’s low adsorption capacity and rate, including through control of the electrode structure,^7,8,19,20^ optimization of the charging protocol,^21–23^ control of redox activity,^9^ and use of a hybrid Zn-ion battery cell.^24^

A key challenge in improving SSA’s CO_2_ adsorption rate and capacity is the lack of clarity around the mechanism of action driving the observed CO_2_ capture behavior.^6^ Prior studies of the SSA mechanism follow the framing put forward in Zhu et al.,^25^ which proposes three potential mechanisms of action that could drive activity (Figure 1b). These mechanisms are differentiated into *gas–solid* and *liquid–solid* mechanisms based on the phase of CO_2_ between gaseous and aqueous phases, and *molecular* and *ionic* mechanisms based on the speciation between CO_2_, HCO_3_^–^, and CO_3_^2–^. These mechanisms are not exclusive, and more than one may be involved in the observed SSA behavior.

In the molecular gas–solid mechanism (Figure 1b, left panel), gaseous CO_2_ is captured in dry activated carbon pores upon charging. Neutron diffraction (ND) measurements of a representative activated carbon material saturated with 1 M NaCl_(aq)_ suggested that up to 80% of the pore volume could remain uninfiltrated by electrolyte, supporting the possible existence of this environment.^26^ CO_2_-pore wall binding strength in the dry pores is proposed to vary upon charging and discharging the activated carbon electrode due to variation in the Fermi level of the material upon charging.^25^ A recent computational study demonstrated the feasibility of this mechanism, with an applied electrical field resulting in a change in CO_2_ binding affinity in a simulated dry carbon cloth adsorbent.^27^ However, the kinetics of CO_2_ capture in SSA processes tend to be slower than expected from gas diffusion.^5^ As a result, the existence of the molecular gas–solid mechanism and whether it contributes significantly to SSA remain an open question.

In the molecular liquid–solid mechanism (Figure 1b, middle panel), aqueous CO_2_ is captured in the electrical double layer (EDL) upon charging. This is proposed to be driven by interactions between charged species and the C=O dipole in the EDL.^25^ A recent study identified that the presence of CO_2_ at an electrode surface in aqueous electrolyte affects capacitance, suggesting the feasibility of the molecular liquid–solid mechanism.^28^ Additionally, other studies have found that the presence of dissolved CO_2_ in a supercapacitor can affect capacitance and the EDL.^29–31^ This suggests the feasibility of a potential molecular liquid–solid mechanism, but conclusive proof of its existence and contribution has not yet been identified.

In the ionic liquid–solid mechanism (Figure 1b, right panel), capture is driven by uptake of aqueous HCO_3_^–^ or CO_3_^2–^. This could occur through inclusion of these anionic species in the EDL of the cathode during charging, which would explain the importance of electrode polarity in SSA measurements.^21^ Combined experimental studies with COMSOL modeling of an SSA module provide evidence for this mechanism,^32^ though this work did not exclude the potential for molecular mechanisms to also contribute to SSA. In a variant of the EDL mechanism, proton uptake at one electrode could also lead to a pH-swing effect, which increases CO_2_ uptake by conversion to HCO_3_^–^. This is supported by several recent preprints that examine the role of pH, and a potential pH-swing, in SSA.^33–35^ As with the molecular liquid–solid mechanism, the ionic liquid–solid mechanism is feasible, but has not been proven to exist or drive SSA; moreover, whether it is driven by double-layer interactions, a pH-swing, or both, remains unclear.

Previous studies that examine the mechanism of SSA possess two key gaps. First, there is a lack of studies that attempt to exclude potential mechanisms as driving SSA. Second, the potential coexistence of multiple mechanisms and how they might interact has not been discussed in detail. In particular, how proposed mechanisms might interact with the CO_2_: HCO_3_^–^ equilibrium has not been significantly discussed. These research gaps motivated us to examine the speciation and phase of dissolved inorganic carbon in SSA electrode/electrolyte systems, to provide mechanistic insight into how SSA occurs.

Solid-state nuclear magnetic resonance (NMR) spectroscopy was chosen as a suitable method to understand the uptake, speciation, and dynamics of dissolved inorganic carbon in model SSA electrode/electrolyte systems. Solid-state NMR spectroscopy is a well-established technique for studying species in electrode–electrolyte interfaces of supercapacitors.^36–39^ Additionally, ^13^C NMR spectroscopy can provide site-specific insight into CO_2_ uptake in porous materials,^40–44^ and ^1^H NMR spectroscopy can be used to probe how water wets activated carbon electrodes.^45^ NMR spectroscopy can also be used to probe exchange processes, including in porous activated carbon materials.^46^

In addition, small-angle neutron scattering (SANS) was identified as an ideal method for understanding wetting in the electrode pores. Contrast-matching SANS for wetting studies exploits the characteristic broad scattering peak observed in the small-angle regime of activated carbons with disordered microporosity.^47^ In a two-phase system, such as the studied carbon and liquid system, the intensity of the micropore scattering feature scales directly with the scattering contrast, (Δ*SLD*_*coh*_)^2^, defined as the squared difference in coherent scattering length density, *SLD*_*coh*_, of the two phases, with *SLD*_*coh*_ determined by the local atomic composition and number density.^48^ For activated carbons with a density (excluding the pores) of approximately 2 g cm^–3^, deuterated water (D_2_O) minimizes contrast with the carbon matrix, with Δ*SLD*_*coh*_ ≈ 0.^49^ Under these conditions, the absence of the nanopore scattering feature indicates complete pore accessibility and full wetting, as demonstrated in previous work.^49–52^ Conversely, a residual scattering signal characteristic of nanopores at the match point can be interpreted as evidence of closed porosity^53^ or incomplete wetting.^54^ This provides additional insight into pore wetting in SSA electrodes beyond previous measurements of pore wetting in SSA using neutron diffraction.^26^

In this work, we applied these methods to understand the phase and speciation of dissolved inorganic carbon in SSA electrodes, and so to understand the mechanism of action. Contrast-matching SANS and ^1^H NMR measurements demonstrated that activated carbon electrodes were completely wetted by electrolyte, and dry pores where the molecular gas–solid mechanism might occur could not be observed, suggesting that SSA must be driven by liquid–solid mechanisms in the examined carbons. Solid-state ^13^C NMR spectroscopy experiments confirmed that SSA is driven by a CO_2(aq)_ or HCO_3(aq)_^–^-driven liquid mechanism in the studied carbons, based on the absence of CO_2(g)_ and CO_3(aq)_^2–^ in the model SSA electrode. CO_2_ and HCO_3_^–^ uptake in the uncharged model electrodes is much higher than SSA uptake from literature results, indicating that SSA uptake represents only a small variation in a large pre-existing pool of dissolved inorganic carbon in the SSA electrodes. Moreover, CO_2_: HCO_3_^–^ exchange was measured and found to be much faster than SSA uptake, suggesting that dissolved inorganic carbon will be stored as both CO_2_ and HCO_3_^–^ regardless of the driving mechanism. These results provide a clearer understanding of the phase and species of dissolved inorganic carbon present in SSA electrodes, and will influence future mechanistic studies and optimization studies of SSA and related CO_2_ capture systems.

## Results

### SANS and NMR Indicate the Molecular Gas–solid Mechanism Does Not Occur

In order to understand whether the hypothesized dry-pore environment required for the molecular gas–solid mechanism (Figure 1b, left panel) exists, contrast-matching SANS studies were carried out on model electrodes made of the activated carbon YP80F soaked in either H_2_O or D_2_O. YP80F is a primarily microporous carbon (see Table S1 and Figure S1 for structural details), which is an effective electrode for SSA.^7,24^ Figure 2a presents the coherent SANS signal, after subtraction of incoherent contributions (Figure S2), for YP80F electrodes that were either dry or soaked in H_2_O and D_2_O. Pure water was used as the liquid phase to model the electrolyte, with the assumption that exclusion of ionic species would not significantly affect the wetting behavior. The SANS data is also presented in a Kratky representation (Figure 2b),^55^ which enhances visibility of pore-related features for easier analysis of wetting. Carbon micropore scattering features (i.e., the broad peak at intermediate q-values, between 0.1 and 0.6 Å^–1^) could be observed in both the dry and H_2_O-wetted carbon (blue and black curves). This was expected, since both air and H_2_O have large Δ*SLD*_*coh*_ contrast values relative to the carbon matrix, resulting in a large micropore peak.^49^ However, in D_2_O (orange curve), the characteristic nanopore scattering peak disappeared completely, suggesting complete wetting of the carbon.^49–52^ If incomplete wetting had occurred, residual dry pockets would have maintained contrast against the carbon matrix and the peak would remain visible.^54^ The absence of this signal therefore demonstrates that the micropores of the YP80F electrode are fully accessible to D_2_O and can be completely wetted with (heavy) water. D_2_O and H_2_O wetting is assumed to be identical, in line with prior studies of H_2_O and D_2_O in activated carbon pores.^45,56^

**Figure 2:**
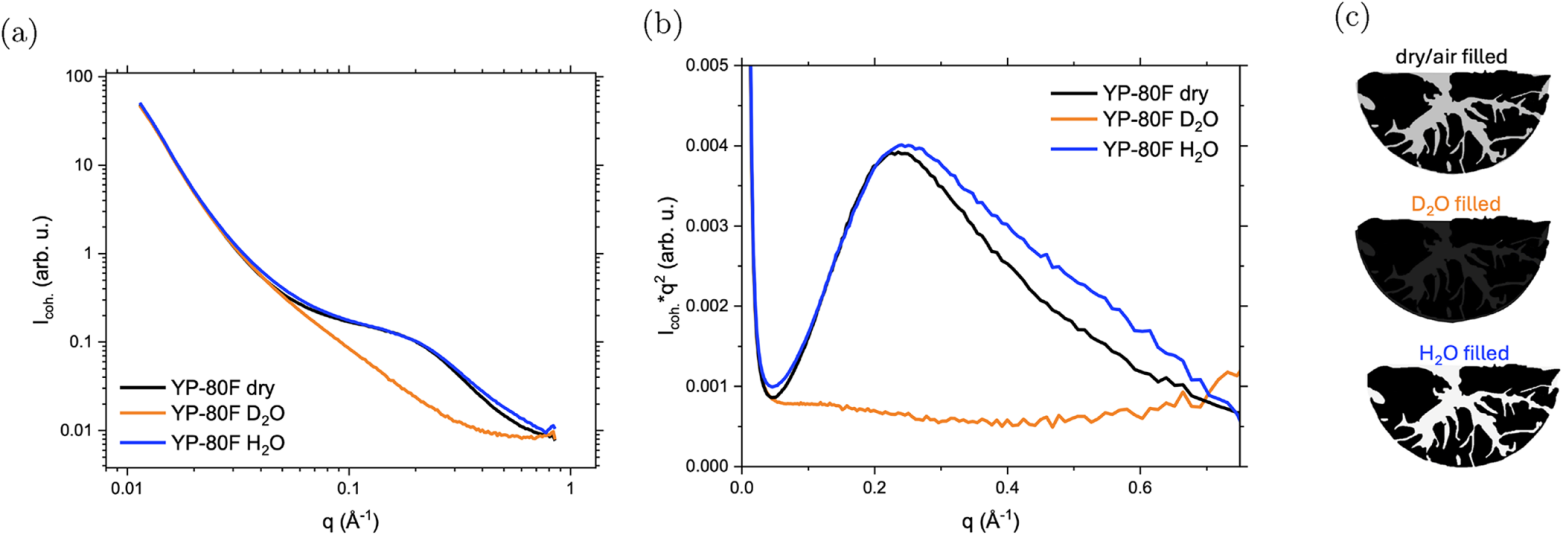
SANS data for dry YP80F (black), and YP80F wetted with D_2_O (orange) and H_2_O (blue). (a) Coherent neutron scattering signal. (b) Kratky representation of the data from (a). This representation enhances the visibility of pore-related features and provides information directly related to chord length and porosity.^55^ (c) Schematic representation of the contrast effect in dry, D_2_O-filled, and H_2_O-filled pore space.

When wetted with H_2_O (Figure 2b, blue curve), enhancement of the nanopore feature was observed due to increased contrast compared to air. Assuming a carbon density of 2 g cm^–3^ and a water density of 1 g cm^–3^, the expected intensity ratio between the H_2_O-soaked and the dry sample is 1.17, while the integrated area of the Kratky plot yields a ratio of 1.13, in good agreement. This comparison notably does not account for a possible variation of the water density within the pores, which may occur since the observed enhancement is not a uniform vertical scaling of the Kratky plot but is accompanied by a subtle change in peak shape. As these changes occur predominantly at higher q-values, corresponding to smaller pores, it suggests that water density within narrower pores may be higher than bulk water density, similar to observations of water density in silica pores^57^ but contrasting with prior observations of water density in carbon pores.^58^

Overall, the SANS results were consistent with the contrast conditions outlined above for complete wetting, illustrated schematically in Figure 2c, and suggested that the YP80F pores were completely saturated with water, with no dry-pore space.

To confirm these observations in the presence of CO_2_, we used NMR spectroscopy to investigate water saturation and CO_2_ speciation in wetted activated carbon films. Samples of YP80F wetted with deionized water were prepared with water:activated carbon (water:AC) mass ratios varying between 0.00 (dry carbon) and 2.01, placed under a ^13^CO_2(g)_ atmosphere, and then examined through ^1^H and ^13^C NMR spectroscopy. Just as in the SANS study, pure water was used as the liquid phase to model the electrolyte, with the assumption that exclusion of ionic species would not significantly affect the wetting behavior. The ^1^H NMR spectra (Figure 3a) reveal three environments, which were assigned to in-pore water (1 to −1 ppm), ex-pore water on the carbon surface (4 to 2 ppm, appearing at mass ratios ≥ 1.33), and bulk-like water (sharp peak at 4.9 ppm, appearing at mass ratios ≥1.54). Separation between the in-pore water and the bulk-like water is driven by a ring current effect caused by the activated carbon, often considered to be a nucleus-independent chemical shift (NICS), which varies with structural factors including the pore size and the ratio of sp^2^:sp^3^ centers.^36,59,60^ The appearance, position, and shape of the ex-pore water environment corresponding to the carbon surface depend on exchange between the in-pore and bulk-like environments, and may also be affected by the activated carbon particle size.^61^ Deconvolution (Figure S3) and integration (Figure 3b) of these peaks identified two wetting regimes. At water:AC mass ratios below 1, the in-pore water integration increased linearly with water loading and no ex-pore or bulk-like water was observed, indicating incomplete pore saturation and the presence of a dry-pore environment. Additionally, water preferentially filled the in-pore environments, with other environments only appearing at higher water loadings. Small changes in the chemical shift and *T*_1_ relaxation times (Table S2) of the in-pore environment were observed as the water:AC mass ratio was increased, which is consistent with prior observations of activated carbon wetting occurring via a multiple-stage mechanism.^62^ Above mass ratios of 1, the in-pore integration plateaued while ex-pore and bulk-like water integrations increased, consistent with complete pore filling. The maximum in-pore loading was therefore in good agreement with the internal pore volume of 1.14 cm^3^ g^–1^ measured with gas sorption (Table S1 and Table S3).

**Figure 3:**
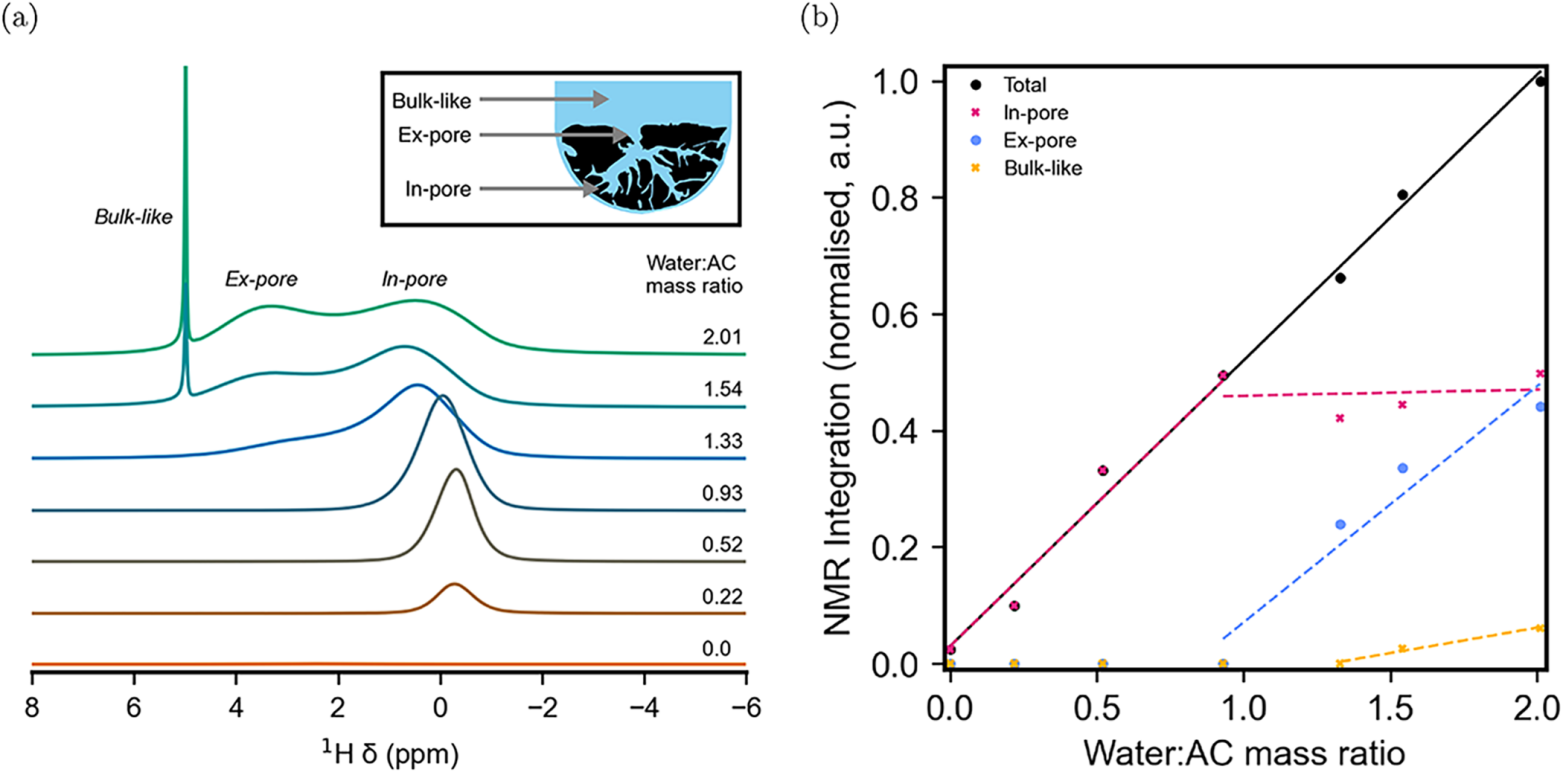
^1^H NMR spectroscopy shows a three-part wetting process in the activated carbon, with the in-pore region filled first and completely saturated at water:activated carbon (water:AC) mass ratios of 1 and above. (a) ^1^H NMR spectra (9.4 T, 5 kHz MAS) of ^13^CO_2(g)_-dosed YP80F model electrodes with varying amounts of water present. Environments for in-pore, ex-pore, and bulk-like water can be observed depending on the loading; peak deconvolutions are provided in the SI. The inset diagram shows where these environments exist in the saturated water:AC system. (b) ^1^H NMR integrations of the total spectrum (black) and the deconvoluted components (pink, blue, and yellow). The change in integration suggests that the in-pore environment is completely saturated at water:AC ratios of ≥1, with ex-pore and bulk water environments appearing at higher water loadings. Linear fits are provided to demonstrate general trends.

These results suggest that all of the internal pore space of the activated carbon is filled with water in an SSA electrode, aligning with our contrast-matching SANS study. The contrast-matching SANS measurements are sensitive to empty pores, and the absence of a signal in the D_2_O-soaked carbon demonstrates complete wetting of the carbon micropores. Meanwhile, the ^1^H NMR measurements examine only the water-filled pores and demonstrate how this wetting process occurs across the in-pore, ex-pore, and bulk-like adsorption environments. As the contrast-matching SANS measurements are less sensitive to exchange, the in-pore environment that they probe includes both the in-pore NMR environment and part of the ex-pore NMR environment. A combination of these techniques therefore allows us to identify the complete wetting of the activated carbon material upon addition of sufficient water to fill the pores.

### ^13^C NMR Spectroscopy Measurements of CO_2_ Speciation

Quantitative ^13^C NMR spectroscopy measurements were also performed to measure dissolved inorganic carbon fractionation, speciation, and concentration in the wet-pore and dry-pore environments in the model ^13^CO_2(g)_-dosed activated carbon/water systems. In the ^13^C NMR spectra (Figures 4a and S4), dissolved inorganic carbon was observed to speciate into up to three species: in-pore CO_2_ (120 ppm), ex-pore/bulk CO_2_ (125 ppm, with very low intensity), and in-pore HCO_3_^–^ (155 ppm). Peaks were assigned based on the literature (more details in the SI).^44,63–68^

**Figure 4:**
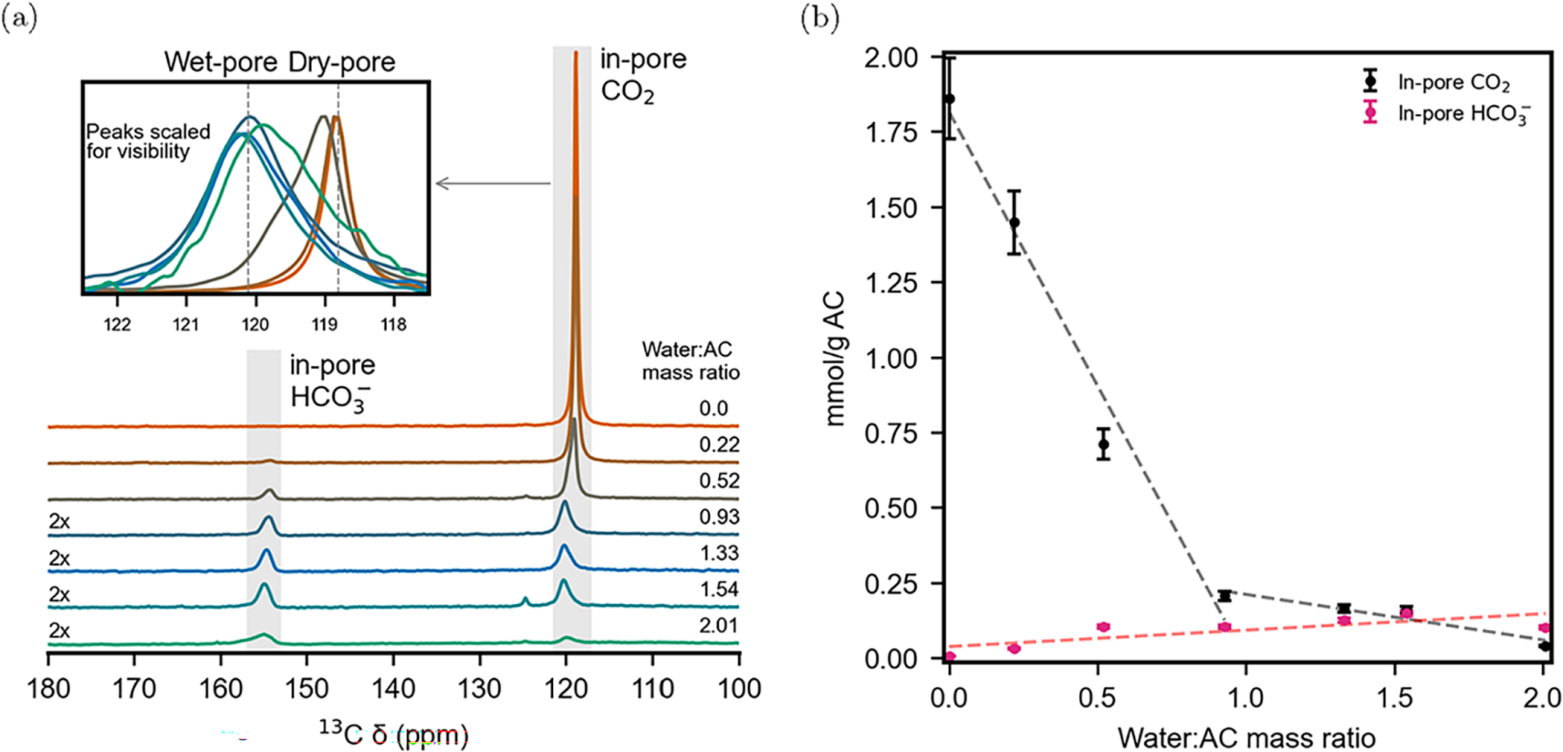
^13^C NMR spectroscopy of the ^13^CO_2_-dosed model SSA electrodes demonstrates how CO_2_ uptake changes as the amount of water present changes. (a) ^13^C NMR spectra (9.4 T, 5 kHz MAS) of ^13^CO_2(g)_-dosed YP80F model electrodes with varying amounts of deionized water present. Main panel: ^13^C spectra normalized to the carbon mass, showing in-pore CO_2_ (120 ppm), ex-pore CO_2_ (125 ppm), and in-pore HCO_3_^–^ (155 ppm). Spectra have been scaled (2x) where necessary to aid visibility. Inset: Close-up of the in-pore CO_2_ peaks, with spectral intensities scaled to enable comparison of chemical shift. A clear distinction is observed between dry-pore CO_2_ capture at 119 ppm (mass ratio 0.0 and 0.22) and wet-pore capture at 120 ppm (mass ratio 0.93 and above). (b) In-pore CO_2_ and HCO_3_^–^ uptake per g of carbon plotted against water:AC mass ratio. In-pore CO_2_ uptake is observed to dramatically decrease as the pores are saturated with water (between mass ratios of 0 and 1 on the *x*-axis), corresponding to the shift from dry-pore to wet-pore regime. Uptake was quantified from peak integration, calibrated to CO_2_ gas sorption experiments, and error bars reflect expected variation in CO_2_ uptake between multiple samples, using the same method described previously.^44^

The chemical shift of the in-pore CO_2_ environment shifted from 119 to 120 ppm as the amount of water inside the system was increased (see the inset of Figure 4a), corresponding to a change from CO_2_ adsorbing in dry pores to CO_2_ dissolving inside in-pore water. This also corresponded to an order-of-magnitude decrease in CO_2_ uptake (Figure 4b), and a change in the ^13^C *T*_1_ relaxation time (Table S4). The coexistence of both of these environments could be observed in the partially wetted 0.52 mass ratio system (Figure S5). CO_2_ uptake was observed to decrease with increasing water mass in the previously identified dry-pore regime (water:AC mass ratio ≤ 1), and then to plateau in the wet-pore regime (water:AC mass ratio ≥ 1). The decreased uptake can be explained by the loss of the dry-pore regions for CO_2_ binding, as these are preferentially filled by water; the absence of the 119 ppm signal in wetted carbons suggests the complete loss of the dry-pore CO_2_ environment.

CO_2_ dissolution in in-pore water in these samples was higher than in bulk water, attributed to an oversolubility effect in the nanoconfined solvent, as reported previously in similar systems.^44^ This mode of wet-pore uptake also explains why CO_2_ uptake decreased more slowly at higher loadings, since uptake is dominated by nanoconfined solvent with little contribution from ex-pore and bulk solvent. The quantity of in-pore HCO_3_^–^ increased with addition of more water to the system (Figure 4b), likely driven by equilibrium effects due to the increased availability of water, which can react to form HCO_3_^–^. The observed decrease in CO_2_ and HCO_3_^–^ uptake for the 2.01 mass ratio system, relative to the other systems in the wet-pore regime, was unexpected and could stem from decreased diffusion of CO_2_ into the pores through the significant amount of bulk water present. Overall, ^13^C NMR spectroscopy measurements indicated that the nature of CO_2_ uptake changes significantly between the dry-pore and wet-pore regimes, suggesting that uptake in the dry-pore regime differs in nature from the wet-pore regime, and that in water-saturated YP80F, no dry-pore environment exists for CO_2_.

These results indicated the implausibility of the molecular gas–solid mechanism for SSA in YP80F, based on the absence of a water-free pore environment where CO_2_ could bind in both the SANS and NMR spectroscopy studies. While the diversity of activated carbon structures suggests that some electrode materials could possess such environments, repeating our NMR spectroscopy experiments on two other activated carbon materials used for SSA showed they also did not possess dry-pore environments (Figure S6). Therefore, in the studied carbon materials, no gas–solid mechanism is possible and SSA behavior is instead driven by liquid–solid mechanisms dominated by dissolved CO_2_ or HCO_3_^–^/CO_3_^2–^. Future studies of novel activated carbon materials that show high SSA activity should test this hypothesis, particularly if fast kinetics are observed, which might require gas–solid behavior to rationalize. Further implications of these results for the mechanism of SSA are examined in the Discussion section below.

### Examination of CO_2_ Dissolution in Different Activated Carbon Model Electrodes

Literature investigations of SSA have demonstrated the importance of the choice of activated carbon material in determining CO_2_ uptake, and suggest that material properties such as mesoporosity, surface area, and wettability could be linked to increased SSA activity.^7,8,19^ In particular, our prior study observed significant differences in CO_2_ uptake between the microporous carbons ACC-10 and ACC-20, the microporous and mesoporous carbons YP50F and YP80F, and the mesoporous carbon CMK-3 (see Table S1 and Figure S1 for porosity characterization).^7^ We therefore applied solid-state NMR spectroscopy measurements to clarify whether the difference in behavior in these different activated carbons could stem from changes in dissolution and fractionation of dissolved inorganic carbon into molecular and/or ionic species for both the in-pore and ex-pore environments. In Figures 5a and S7, the ^13^C NMR spectra for five different activated carbons (ACC-10, ACC-20, YP50F, YP80F, and CMK-3) are shown, mixed with sufficient 1 M Na_2_SO_4(aq)_ as electrolyte to saturate the carbon pores, and dosed with ^13^CO_2_, replicating the systems used in this prior study.^7^ Separate environments for CO_2_ (around 120 ppm) and HCO_3_^–^ (around 160 ppm) were observed in all five spectra, indicating that CO_2_ dissolved into the electrolyte and formed HCO_3_^–^ in all the studied systems. These environments in the ACC and YP carbon samples showed paired peaks, separated by about 5 ppm, reflecting the in-pore and ex-pore populations of CO_2_ and HCO_3_^–^ due to the activated carbon-induced ring current effect. Additionally, the position of the peak at 160 ppm indicated that the environment was entirely HCO_3_^–^, and no significant population of CO_3_^2–^ existed, based on NMR titration of a HCO_3_^–^/CO_3_^2–^ mixture (Figure S8).^68^ In contrast, in the CMK-3 spectrum, the in-pore and ex-pore peaks could not be resolved. This could be due to fast exchange or from peak overlap. This also prevented unambiguous assignment of the peak at 160 ppm, though it likely reflected in-pore HCO_3_^–^ based on measurement of the ^1^H peak separation due to the NICS (Figure S9). Quantification of CO_2_ and HCO_3_^–^ uptake (Figure 5b and Table S5) revealed that the choice of carbon significantly affected speciation, but not the total dissolved inorganic carbon. As with the YP80F/water system, dissolved inorganic carbon uptake was significantly higher than would be expected for equivalent volumes of 1 M Na_2_SO_4_, due to nanoconfinement-induced oversolubility (Table S5).^44,69^ Despite their diverse structures, origins, and preparation methods, all carbons took up similar quantities of dissolved inorganic carbon, with in-pore CO_2_ and HCO_3_^–^ observable in significant quantities in all spectra. Qualitative analysis of the environments observed in the ^13^C NMR spectra therefore suggested the feasibility of both CO_2_-driven and HCO_3_^–^-driven liquid–solid mechanisms of SSA.

**Figure 5:**
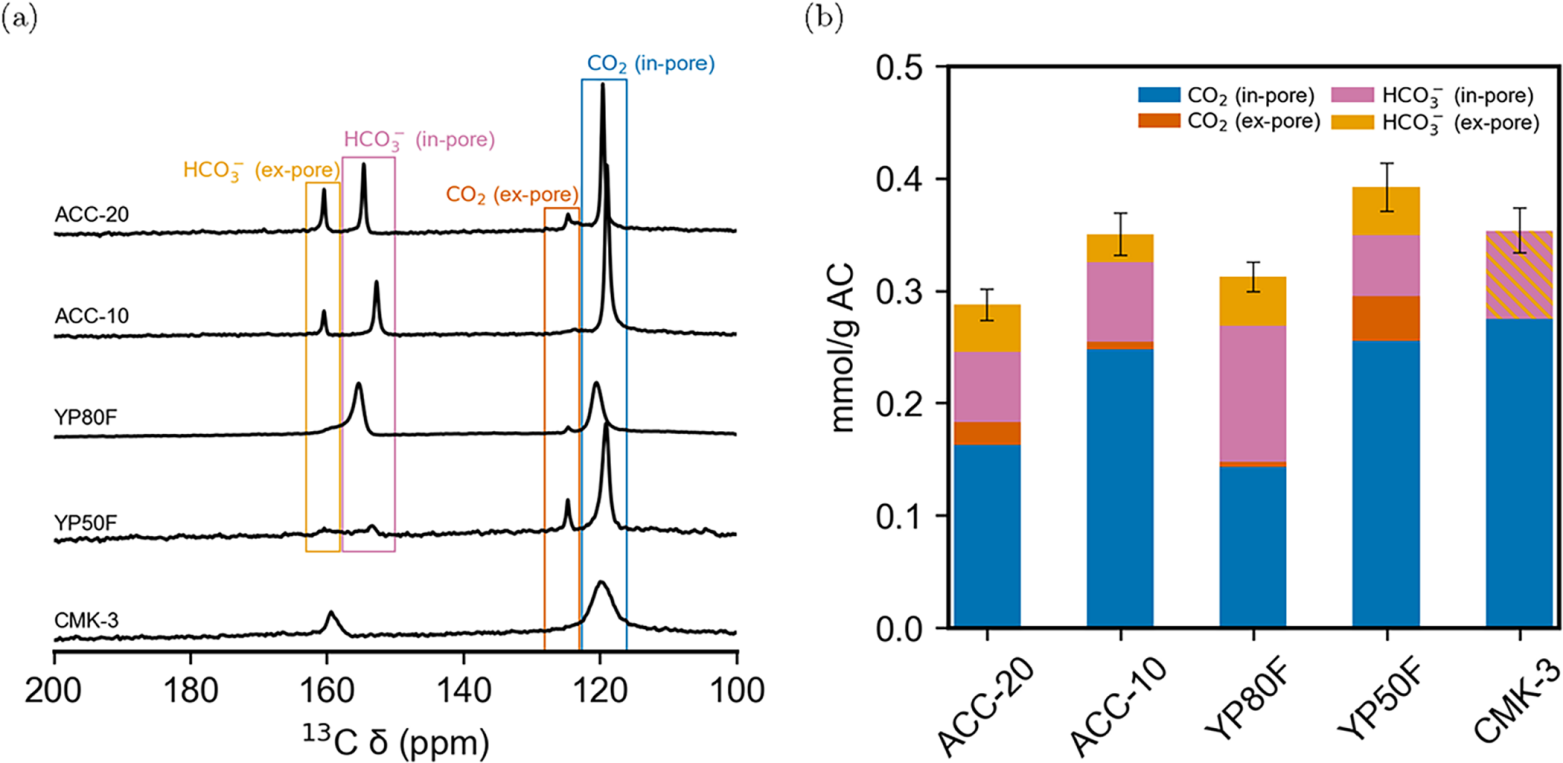
Quantitative ^13^C NMR spectroscopy of ^13^CO_2_-dosed activated carbon/1 M Na_2_SO_4(aq)_ systems reveals consistently high initial uptake but variable speciation. (a) ^13^C NMR spectroscopy (9.4 T, 5 kHz MAS) of dosed samples with a variety of different activated carbons, all saturated with 1 M Na_2_SO_4(aq)_ as the model electrolyte. The ex-pore and in-pore HCO_3_^–^ and CO_2_ environments used for integration and quantification in part (b) are labeled. The peak at 160 ppm in CMK-3 was tentatively assigned to in-pore HCO_3_^–^, but could not be conclusively assigned (see main text). (b) CO_2_ uptake per gram of carbon in the studied activated carbon/1 M Na_2_SO_4_ systems, divided into in-pore and ex-pore CO_2_ and HCO_3_^–^. Uptake was quantified from peak integration, calibrated to CO_2_ gas sorption experiments, and error bars reflect expected variation in CO_2_ uptake between multiple samples, using the same method described previously.^44^ The hashed color for HCO_3_^–^ in CMK-3 reflects the uncertain assignment in this system.

Given the potential role of in-pore CO_2_ and HCO_3_^–^ in SSA, we then sought to compare initial uptake in these environments to the electrochemically driven CO_2_ uptake during charging. Figure 6a shows the in-pore CO_2_ and HCO_3_^–^ uptake measured by NMR alongside literature-reported CO_2_ uptake for each carbon in an SSA module using 1 M Na_2_SO_4(aq)_ electrolyte at fast (150 mAg^–1^) and slow (10 mAg^–1^) charging rates.^7^ The reported SSA uptake in these systems represented at most a 30% swing in the dissolved inorganic carbon concentration present inside the uncharged electrode. The SSA uptake was correlated with the initial (NMR-derived) HCO_3_^–^ uptake (Figure 6b), which suggested that the position of the CO_2_:HCO_3_^–^ equilibrium may influence SSA, aligning with recent studies of electrolyte pH effects on SSA.^33,35^ Other initial uptake parameters did not appear to correlate with SSA uptake (Figure S10). Due to the many degrees of freedom in examining correlations in this system, and the relatively large error observed in these measurements, the proposed correlation of HCO_3_^–^ uptake to SSA performance requires further study.

**Figure 6:**
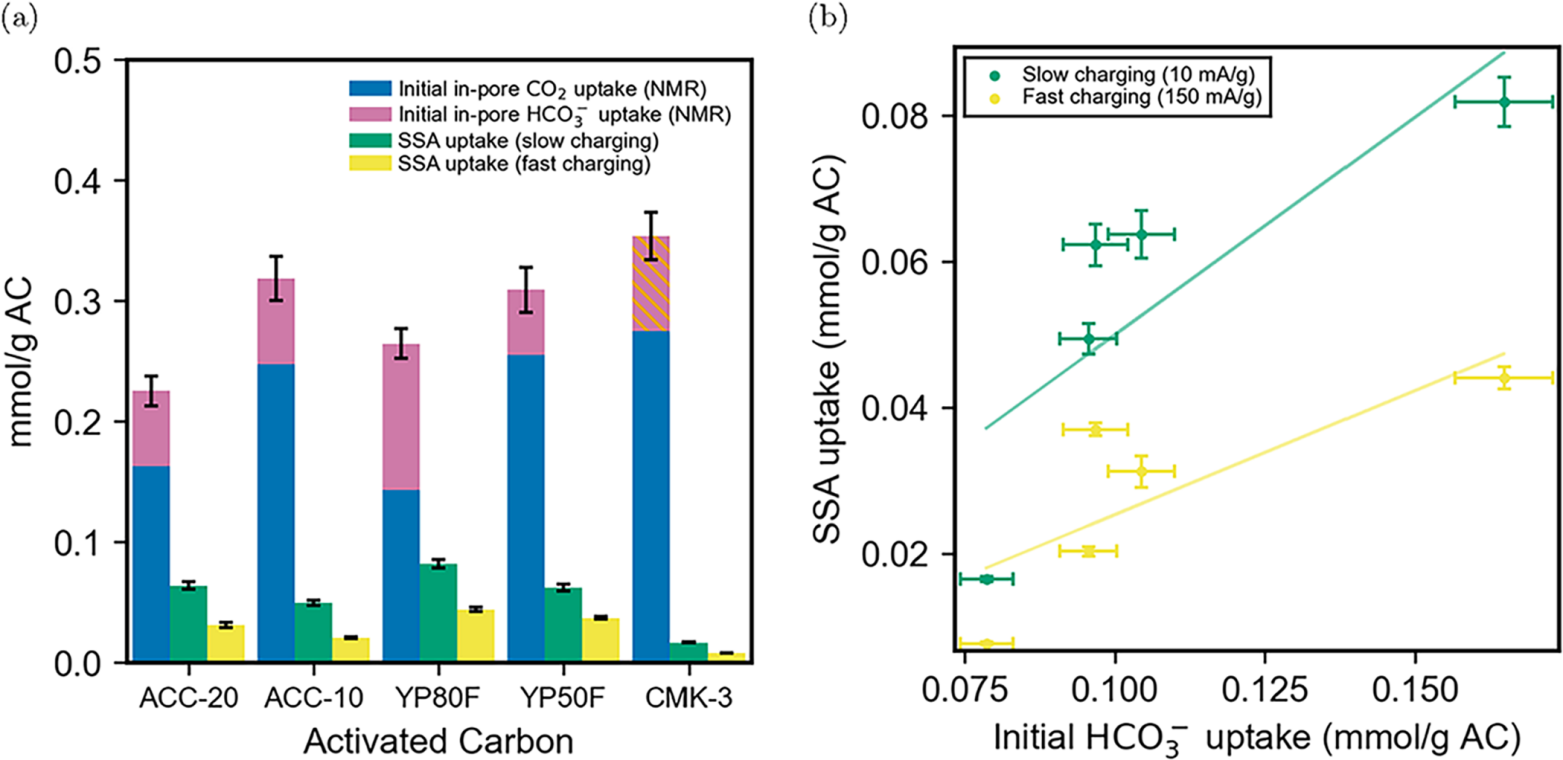
Comparison of NMR-measured initial uptake to SSA-driven uptake reveals that SSA reflects a small variation in a large pre-existing pool of dissolved inorganic carbon. (a) Comparison of initial in-pore CO_2_ and HCO_3_^–^ uptake in model electrodes, measured through NMR, to SSA uptake measured in Xu et al.^7^ (b) Plotting SSA uptake from Xu et al.^7^ against NMR-measured initial HCO_3_^–^ uptake suggests a potential correlation, expressed in lines of best fit (with *R* = 0.81 for slow charging and *R* = 0.78 for fast charging). In both parts (a) and (b), error bars on initial uptake reflect expected variation in CO_2_ uptake between multiple samples, as described previously.^44^ Error bars for SSA uptake come from the literature.

### Examining CO_2_:HCO_3_^–^ Exchange in SSA Processes

To better understand the behavior of HCO_3_^–^ and CO_2_ in an SSA electrode, ^13^C NMR spectroscopy was used to probe the physical and chemical exchange of these species. CO_2_:HCO_3_^–^ exchange has been previously incorporated into the modeling of SSA,^32^ but it has not been empirically measured in an SSA system. We examined exchange through 2D exchange spectroscopy (EXSY) measurements (Figure 7a) of the ^13^CO_2(g)_-dosed ACC-20/1 M Na_2_SO_4(aq)_ system, since its NMR spectrum contained narrower peaks than other studied carbons. In this experiment, peaks on the diagonal reflect nuclei that remain in the same environment through the mixing time, and so should be similar to the peaks of the 1D experiment (top projection of Figure 7a). Slow exchange between two environments can be observed through the appearance of cross-peaks off the diagonal, whose magnitude depends on the exchange rate and the mixing time. In the 2D ^13^C spectrum in the ACC-20/1 M Na_2_SO_4(aq)_ system, these cross-peaks could be observed for physical exchange between in-pore and ex-pore regions, and for chemical exchange between in-pore CO_2_ and HCO_3_^–^ (Figure 7a, main panel). Notably, peaks could be observed corresponding to exchange between ex-pore and in-pore CO_2_, despite the lack of an ex-pore CO_2_ diagonal peak; this was due to a combination of fast exchange and fast relaxation in the ex-pore environment (Table S6), which both reduce the signal for the diagonal peak. The cross-peak signals for physical exchange between in-pore and ex-pore environments were stronger than the cross-peak observed for chemical exchange between CO_2_ and HCO_3_^–^ (Figure S11), indicating that chemical exchange was slower than physical exchange between environments. ^13^C exchange NMR spectroscopy measurements therefore revealed the complexity of the SSA electrode/electrolyte system, with both physical exchange between pores and chemical exchange between ionic and molecular species being observable on the NMR time scale.

**Figure 7:**
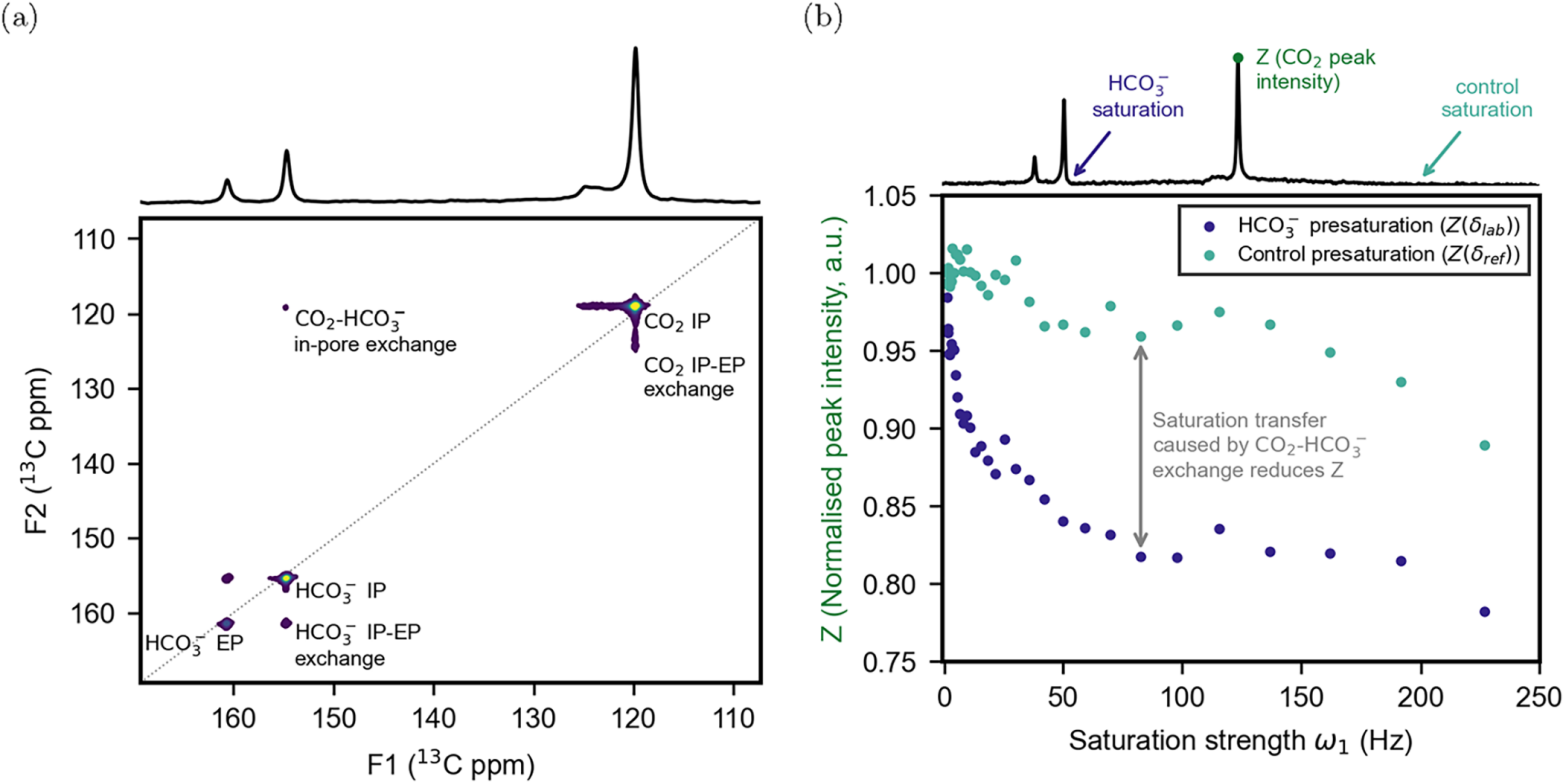
(a) 2D ^13^C- ^13^C EXSY (9.4 T, 5 kHz MAS, 50 ms mixing time) spectrum of ^13^CO_2_-dosed ACC-20 + 1 M Na_2_SO_4_ demonstrates physical exchange between in-pore (IP) and ex-pore (EP) environments, and chemical exchange between in-pore CO_2_ and in-pore HCO_3_^–^. The projection at the top of the figure shows the 1D ^13^C spectrum for the same sample. The dotted line shows the diagonal F1 = F2; peaks on this diagonal correspond to the 1D peaks. (b) ^13^C QUESP (9.4 T, 5 kHz MAS) plot of ^13^CO_2_-dosed ACC-20 + 1 M Na_2_SO_4_ demonstrates chemical exchange between in-pore HCO_3_^–^ and CO_2_.

To further understand the relationship between CO_2_ and HCO_3_^–^, we performed “quantification of exchange through saturation power” (QUESP) NMR experiments to quantify the slower chemical exchange rate through saturation transfer.^70–72^ QUESP experiments were chosen over quantitative EXSY exchange measurements as they were affected less by the fast relaxation observed, enabling recording of more fitting points. In the QUESP experiment, the intensity of the primary peak (at frequency ω) is measured, while a second labeled peak (at intensity Δ*ω*) is saturated at variable saturation strength (ω_1_) to suppress the NMR signal.

In this work, the intensity of the in-pore CO_2_ peak was measured while the in-pore HCO_3_^–^ environment was labeled through variable saturation (Figure 7b), in order to measure the exchange rate *k*_*b*_ for the equilibrium ${\mathrm{CO}}_{2}\overset{k_{a}}{\underset{k_{b}}{⇌}}{\mathrm{HCO}}_{3}^{-}$ . *k*_*a*_ and *k*_*b*_ are pseudo-first-order reaction constants incorporating the concentrations of water, OH^–^, and H_3_O^+^. Saturation of the labeled peak (at frequency Δ*ω*) results in enhanced reduction of the primary peak (at frequency ω) if the peaks are in exchange; this decrease is quantified by the parameter *Z*(Δ*ω*, ω_1_) = *M*_*sat*_(Δ*ω*, ω_1_)/*M*_0_, where *M*_*sat*_(Δ*ω*) is the primary peak’s intensity upon saturation of the signal at frequency Δ*ω* with saturation strength ω_1_, and *M*_0_ is the primary peak’s intensity in the unsaturated spectrum. To extract an exchange rate from the QUESP experiment, *Z* is recorded at a variable saturation strength ω_1_, first in the labeled scan for Δ*ω* = δ_*lab*_ (Figure 7b, purple), and then in the reference scan for Δ*ω* = δ_*ref*_ = −δ_*lab*_ (Figure 7b, green), which is used to account for spillover saturation of the primary peak that does not stem from exchange. The ratio between *Z*(δ_*lab*_) and *Z*(δ_*ref*_) can then be fitted to a model to extract an exchange rate (Figure S12).

For the ACC-20/1 M Na_2_SO_4(aq)_ system shown, *k*_*b*_ was fitted to be 0.22 ± 0.12 s^–1^, and *k*_*a*_ was fitted to be 0.084 ± 0.046 s^–1^ (95% interval). CO_2_ hydration and HCO_3_^–^ are well-studied processes in bulk aqueous systems, and exchange constants for these reactions are known and were compared to our calculated figures.^73,74^ *k*_*b*_ is the exchange rate expected in bulk solution at approximately pH 5.8, and *k*_*a*_ is the exchange rate expected in bulk solution at approximately pH 8.6 (based on eqs 6 and 7 in the SI). These calculated *k*_*a*_ and *k*_*b*_ values are therefore on similar orders of magnitude for bulk CO_2_:HCO_3_^–^ exchange in close-to-neutral aqueous solution, as expected for the system. However, they are clearly not consistent with each other. The observed difference is due to the well-established tendency for micropore-confined aqueous systems to show different behavior from the bulk, including changes to water density,^57,58^ water reactivity and autoionization,^75,76^ and CO_2_:HCO_3_^–^ hydration.^77,78^ It is expected that these changes would significantly affect CO_2_:HCO_3_^–^ exchange inside carbon pores, explaining the apparent inconsistency. ^13^C QUESP NMR spectroscopy therefore provides a useful tool to observe and quantify CO_2_: HCO_3_^–^ exchange in an SSA system.

To further understand how our measured rates of CO_2_:HCO_3_^–^ exchange would interact with SSA activity, we modeled a simple three-site SSA process involving CO_2(aq)_, HCO_3(aq)_^–^, and CO_2(g)_ (Figure 8a). Using the NMR-derived initial concentrations for [CO_2(aq)_] and [HCO_3(aq)_^–^], and the NMR exchange measurements to estimate *k*_*a*_ and *k*_*b*_, exchange was modeled between CO_2_ and HCO_3_^–^ in the SSA module. In-pore and ex-pore populations were combined in this model due to the rapid exchange observed between those environments in the EXSY experiment. We then introduced an irreversible zero-order SSA process, defined by the zero-order exchange parameter *k*_*SSA*_, to model the capture of gaseous CO_2_ via either a molecular or ionic mechanism. *k*_*SSA*_ was set to 349.34 mmol kg^–1^ h^–1^, reflecting the highest reported rate of CO_2_ capture in Xu et al.,^7^ which is faster than the majority of reported SSA processes. Figure 8b shows the modeled concentration changes over time for CO_2_ and HCO_3_^–^, as well as the net CO_2_ capture from CO_2(g)_, in this model for either a molecular or ionic mechanism of capture. Concentrations for CO_2_ and HCO_3_^–^ were effectively identical in this model, regardless of the mechanism of action. From this simple model, it was clear that the CO_2_: HCO_3_^–^ exchange is much faster than supercapacitive CO_2_ capture. This indicated that mechanisms of SSA must account for the presence of both CO_2_ and HCO_3_^–^ when identifying the driving force of capture, as these populations will be in rapid exchange. Figure 8c presents a more realistic representation of SSA behavior under these mechanisms, reflecting the likely reality of in-pore storage of combined CO_2_ and HCO_3_^–^ during an SSA experiment. A simple model of an SSA process, informed by empirical NMR measurements of exchange between CO_2_ and HCO_3_^–^, therefore suggested that SSA captures CO_2_ as both a molecular and ionic species.

**Figure 8:**
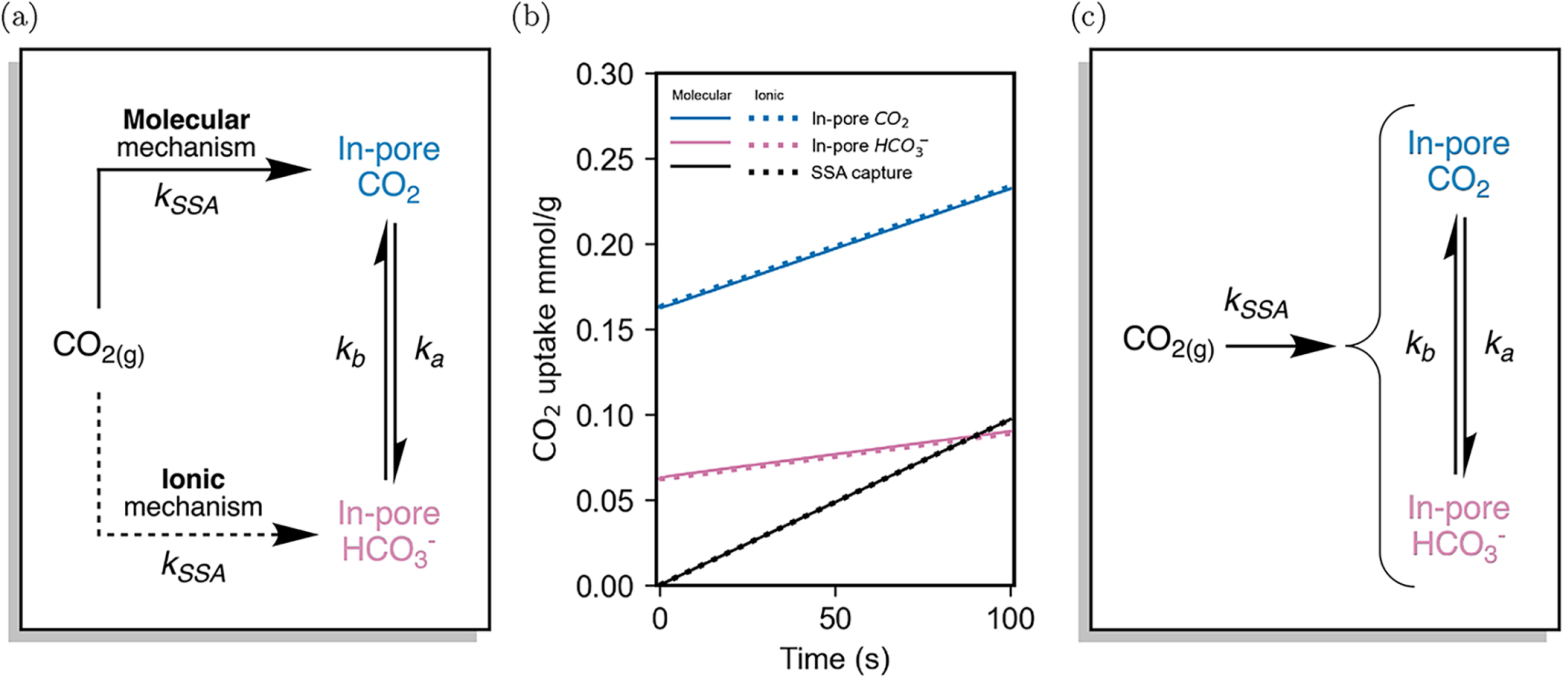
(a) Model showing how proposed molecular and ionic mechanisms of SSA would interact with exchange between in-pore CO_2_ and HCO_3_^–^. (b) CO_2_ and HCO_3_^–^ quantities inside an SSA electrode during an idealized SSA charging process were calculated using the model from (c), *k*_*a*_ and *k*_*b*_ from NMR measurements, and *k*_*SSA*_ from Xu et al.,^7^ (taking the highest observed rate of capture as a limiting value). This model suggests that captured in-pore CO_2_ and HCO_3_^–^ would likely coexist. (c) More representative model of the proposed behavior of a liquid–solid mechanism of SSA, which reflects the observed in-pore exchange between CO_2_ and HCO_3_^–^.

Several assumptions were made in this model. First, ex-pore populations were combined with in-pore populations for CO_2(aq)_ and HCO_3(aq)_^–^. This was justified on the basis that ex-pore population is less than the in-pore population, and that in-pore:ex-pore exchange was observed to be faster than CO_2(aq)_:HCO_3(aq)_^–^ exchange in the 2D EXSY environment. Second, the concentration of OH^–^ (and H_3_O^+^) was taken as a constant when calculating *k*_*b*_, but could vary across the modeled SSA process. The activated carbon surface has been previously observed to buffer the electrolyte pH,^37^ but further investigation into the effects of pH on SSA should be undertaken to confirm this assumption. Third, the ionic liquid–solid mechanism was modeled as CO_2(g)_ → HCO_3(aq)_^–^, whereas some literature mechanisms^6,21,32^ instead propose a CO_2(g)_ → CO_2(aq)_ → HCO_3(aq)_^–^ pathway, which corresponds to the molecular liquid–solid mechanism modeled here; however, this does not alter the central conclusion that both the molecular liquid–solid and ionic liquid–solid mechanisms result in combined CO_2_/HCO_3_^–^ uptake.

## Discussion

### Evidence against the Molecular Gas–Solid Mechanism

The molecular gas–solid mechanism has been considered as a potential driving force for SSA since it was first reported.^5^ However, the rate of CO_2_ capture has proven to be slower than would be expected for the molecular gas–solid mechanism, leading to the proposal of alternative liquid-phase mechanisms.^5,79^ Our NMR spectroscopy and SANS experiments provided confirmatory evidence for these liquid-phase mechanisms by excluding the possibility of the molecular gas–solid mechanism from occurring in the studied SSA electrodes YP80F, ACC-20, and ACC-10, due to the lack of a dry-pore environment observed across three different activated carbons examined in this study.

These results notably conflict with a prior study, which used neutron diffraction (ND) measurements to study D_2_O saturation of pores in a different activated carbon material and reported that significant pore space remained dry.^26^ The variation in results likely stems from a combination of differences in measurement methodology and the choice of the material. ND in the wide-angle regime provides information on the overall carbon and liquid (D_2_O or H_2_O) ratio through the structure factor, but it is only sensitive to atomic-scale correlations and therefore does not resolve whether D_2_O actually penetrates and occupies the micropore space. Contrast-matching of pore filling requires access to the associated pore scattering features, which arise in the small-scattering regime and are therefore probed by SANS, as carried out in this work, not the wide-angle regime investigated with ND. The sample may also have contributed, as the prior study used a different activated carbon to YP80F and pelletized the material, while our study used a thin film to better reflect the sample morphology in SSA electrodes.

Whether our results are generalizable for all activated carbon/electrolyte systems used in SSA remains an open question. As noted in prior literature,^5^ the kinetics of CO_2_ capture in SSA are slower than would be expected for a gas–solid system, suggesting it does not occur in other systems. Dry pores could also potentially be observed if insufficient electrolyte was used to saturate the activated carbon pores or if the activated carbon used contained extremely hydrophobic pores, and such setups could be used to probe the feasibility of the molecular gas–solid mechanism as a CO_2_ capture mechanism further.

### Molecular and Ionic Contributions to the Liquid–solid Mechanism

Considering the liquid–solid mechanisms, a separate question is whether supercapacitive CO_2_ capture during charging is driven by the behavior of CO_2(aq)_ (molecular liquid–solid) or HCO_3(aq)_^–^ (ionic liquid–solid). Results in this work demonstrated that both CO_2_ and HCO_3_^–^ are present in model SSA electrodes in large quantities relative to supercapacitive CO_2_ capture capacity, indicating the feasibility of either species causing the observed SSA behavior. Furthermore, NMR measurements of exchange indicated that in-pore CO_2_ and in-pore HCO_3_^–^ interconvert faster than the rate of SSA capture and release. Therefore, in both the molecular liquid–solid and ionic liquid–solid mechanisms, dissolved inorganic carbon could be stored as a combination of both CO_2(aq)_ and HCO_3(aq)_^–^. This suggests that a mechanism which affects either CO_2_ or HCO_3_^–^ is feasible, in line with evidence in the literature supporting both the molecular liquid–solid mechanism^28^ and the ionic liquid–solid mechanism.^21,32^ Additionally, the NMR measurements of exchange suggest that the rate-limiting step for such a mechanism is not CO_2_:HCO_3_^–^ exchange, meaning that kinetic limitations to SSA must stem from another rate-limiting step. One possible explanation is mass-transport limitations, which would be especially notable if migration through the tortuous and microporous activated carbon electrode is necessary; this behavior could explain the importance of combined microporosity and mesoporosity observed in prior work.^7^

The rapid exchange observed in the CO_2_:HCO_3_^–^ equilibrium, and the expected pH-dependence of the equilibrium, also suggest that pH could play a significant role in either controlling or driving SSA. A recent work proposes an electrochemical pH-swing mechanism, which could be driven by reactions between H^+^ and the carbon surface^34^ and/or enhanced water dissociation under an electrical field.^35^ In such a process, HCO_3_^–^ may also be stored in the bulk electrolyte, rather than inside the pores. Such a mechanism could also explain the correlation between SSA uptake and initial HCO_3_^–^ formation observed in this work (Figure 6b), since the presence of large quantities of bicarbonate would suggest a more basic electrolyte environment, which might be more favorable for SSA. A pH-swing process also suggests the possibility of alternate rate-limiting steps to CO_2_:HCO_3_^–^ exchange, such as whatever process leads to the local changes in H^+^ and OH^–^ concentration. The NMR spectroscopy methods outlined in this work will provide an effective method to probe dissolved inorganic carbon speciation inside and outside pores under a potential pH-swing, and further research on this topic is ongoing in our laboratory.

## Conclusions

Our findings demonstrated that supercapacitive CO_2_ capture is driven by the behavior of dissolved CO_2(aq)_ and/or HCO_3_^–^, with no contribution from CO_2(g)_ in the studied systems. Contrast-matching SANS measurements of pore wetting found that the pores in a YP80F activated carbon electrode were completely wetted upon saturation by D_2_O. This was confirmed by solid-state NMR spectroscopy studies of multiple model activated carbon electrodes after dosing with ^13^CO_2_, which demonstrated that CO_2_ uptake in wetted electrodes occurred through formation of CO_2(aq)_ and HCO_3(aq)_^–^, with no gas-phase CO_2(g)_ adsorption occurring. Examination of a range of ^13^CO_2(g)_-dosed activated carbon electrodes through NMR spectroscopy demonstrated that the uncharged system contained large reservoirs of dissolved inorganic carbon, and that SSA capture and release represent a relatively small change in this reservoir. In particular, SSA capture appeared to be correlated with the amount of HCO_3_^–^ present prior to initial charging. Exchange measurements suggested that in-pore CO_2_ and HCO_3_^–^ exchanged very quickly relative to the rate of SSA capture. This suggested that the molecular liquid–solid and ionic liquid–solid mechanisms would produce very similar in-pore environments, with both molecular and ionic species present.

These results provided significant insight into the nature of the in-pore environment of the porous electrodes used in an SSA cell prior to charging. Work is underway to extend these insights by understanding how the initial CO_2_ and HCO_3_^–^ populations evolve upon charging and discharging of the SSA cell. Further research will also test whether the observed correlation between initial HCO_3_^–^ uptake and SSA activity is a viable method for identifying high-performance activated carbon materials for SSA. Finally, a recent work on the possibility of a pH-swing mechanism of SSA suggests the importance of understanding how the speciation and exchange behaviors observed might change as in-pore and ex-pore pH vary. These findings will therefore influence further mechanistic studies of SSA and the design and optimization of SSA modules.

## Experimental Methods

## Methods

### Materials

Activated carbon cloths ACC-5092-10 (ACC-10) and ACC-5092-20 (ACC-20) were purchased from Kynol. Activated carbon cloths were washed with approximately 500 mL of deionized water (per 5 g of carbon) and dried in a vacuum oven at 90 °C for 24 h before storage under atmospheric conditions.

YP80F, YP50F, and CMK-3 were made into free-standing carbon films by mixing 95 wt % activated carbon with 5 wt % polytetrafluoroethylene binder (Sigma-Aldrich, 60 wt % dispersion in water) in ethanol. The film was manually rolled to 0.25 mm thickness and dried in a vacuum oven at 90 °C for 24 h to remove residual water and ethanol before storage under atmospheric conditions.

Sodium sulfate (anhydrous, ≥99%) salt was purchased from Fisher Scientific and then made into a 1.00 M aqueous solution with deionized water.

^13^C-enriched CO_2_ gas was purchased from Cambridge Isotope Laboratories, with 98 at. % ^13^C enrichment.

### Preparation and ^13^CO_2_ Dosing of Model Uncharged SSA Electrodes for NMR Spectroscopy

For each activated carbon/solvent sample, the activated carbon (either cloth or film) was cut into small pieces, and then an approximately 10 mg sample was weighed and placed in a vial. Solvent (either 1 M Na_2_SO_4(aq)_ or deionized water) was then added to achieve the desired mass/volume ratio between the activated carbon and the solvent. The vial was sealed and left for 5 min to allow solvent to saturate the sample and then packed into a 3.2 mm rotor as quickly as possible to minimize evaporation of the solvent. Samples and vessels were weighed before and after the addition of solvent and packing to quantify any loss of solvent due to evaporation.

Each sample was briefly evacuated for 1 min under a static vacuum in a home-built gas manifold, as described previously.^80^ Samples were then dosed with ^13^CO_2(g)_ at room temperature at an initial atmosphere of 0.7 bar for 30 min, before sealing the rotors inside the gas manifold with a mechanical plunger.

### NMR Spectroscopy Experiments

Solid-state NMR spectroscopy experiments were carried out with a Bruker Avance Neo spectrometer in a Bruker 3.2 mm HXY triple resonance probe. Measurements were carried out at a magnetic field strength of 9.4 T, corresponding to a ^1^H Larmor frequency of 400.1 MHz. All spectra were acquired with a 90° pulse-acquire sequence at an MAS speed of 5 kHz, at which speed we observe resolved spectra while avoiding excess frictional heating or centrifugation.^81,82^ The 90° pulse length was optimized for each sample. Recycle delays were set to >5*T*_1_ for the CO_2_-derived peaks for each sample to ensure measurements were quantitative, based on measurements of *T*_1_ through either inversion recovery experiments or FLIPS.^83^ Measurements were not quantitative for the activated carbon background peaks. ^13^C and ^1^H NMR spectra were referenced relative to the ^13^C CH resonance of adamantane at 37.78 ppm as a secondary reference, using Ξ­(^13^ *C*) = 25.145 972% to reference the ^1^H spectra.^84–86^

1D NMR spectra were deconvoluted and fitted using ssNake.^87^ The fitting of each spectrum was repeated at least 3 times. For each fitting, different initial peak shapes were applied and then varied freely to achieve the optimal fitting.

Solution-state NMR spectroscopy experiments were carried out in a Bruker Avance Neo spectrometer operating at a magnetic field strength of 14.1 T (600 MHz 1H Larmor frequency). A Bruker 5 mm F/P/C–H QNP probe was used. ^13^C solution-state experiments were referenced to the CH_3_ peak of ethanol at 18.4 ppm on the tetramethylsilane (TMS) scale,^88^ with a nonquantitative recycle delay.

### NMR Spectrum Assignment

#### ^1^H Spectrum Peak Assignment

Examination of the ^1^H NMR spectra of activated carbon/electrolyte systems showed either up to three peaks, depending on the choice of carbon and the electrolyte loading. ^1^H spectra of YP80F and YP50F showed up to three overlapping peaks at approximately 5, 3, and 0 ppm, which were assigned to bulk, ex-pore, and in-pore water, respectively, with peak separation ascribed to a nucleus-independent chemical shift (NICS) effect as well as exchange effects in line with the literature.^36,61,81^ The bulk peak (5 ppm) was characteristically sharp, and its chemical shift varied very little between systems. The ex-pore and in-pore water peaks varied in shape and size more significantly among systems. At low loadings, only the in-pore peak is observed. As the amount of electrolyte is increased, first the ex-pore and then the bulk peak are observed.

^1^H spectra of ACC-10 and ACC-20 showed up to two visible peaks at approximately 4 and −1 ppm, which were assigned to ex-pore and in-pore water separated by a NICS effect.^38^ Only the in-pore peak (at −1 ppm) is seen at low electrolyte loadings. The peak at 4 ppm appears to be made up of two overlapping peaks, which likely represent an ex-pore and bulk-like environment (as observed in YP80F).

^1^H spectra of CMK-3 showed two overlapping peaks at 4 and 2 ppm, which were assigned to an ex-pore and an in-pore/exchange peak. This behavior was ascribed to the large pore size of CMK-3, which both increases exchange in and out of the pores and decreases the size of the NICS.^36,81^ Variable-volume loading with electrolyte was not performed, so the loadings at which these peaks appear were not identified.

#### ^13^C Spectrum Peak Assignment

After dosing with ^13^CO_2(g)_, uptake of CO_2_ and formation of HCO_3_^–^ are observed in the ^13^C NMR spectrum. Details of spectrum assignment have been described previously,^44^ and are repeated with minor adjustments below.

In the YP and ACC carbons, up to four sharp peaks were observed after dosing with ^13^CO_2(g)_. Peak shifts varied slightly depending on the choice of activated carbon and the choice and quantity of solvent (±2 ppm from stated values below). CO_2_ environments are observed at 125 and 120 ppm, corresponding to ex-pore and in-pore environments separated by a NICS. Ex-pore CO_2_ was assigned based on literature studies, which show chemical shifts of 124–126 ppm in both the aqueous and gaseous state,^63–66,89^ and in-pore CO_2_ was assigned based on these literature shifts alongside a NICS effect, and based on the measurement of the dry ^13^CO_2(g)_-dosed carbon. Determining whether the ex-pore CO_2_ peak is aqueous or gaseous was infeasible due to the small intensity of the peak in most spectra. In some ^13^C NMR spectra, the ex-pore CO_2_ peak’s shape suggested the potential for a two-peak fitting, which could correspond to bulk and ex-pore CO_2(aq)_ or aqueous and gaseous CO_2_, but deconvolution was not carried out due to the potential for overfitting on such a small signal. HCO_3_^–^ environments are observed at 160 ppm (ex-pore) and 155 ppm (in-pore) in wetted samples.^64,67^ Rapid HCO_3_^–^/CO_3_^2–^ exchange means that this environment also represents any CO_3_^2–^ present.^68^

Broad background peaks can also be observed in measured ^13^C spectra, spanning at least the range 200–50 ppm. These can be clearly seen in ^13^C spectra of the activated carbon measured without ^13^CO_2_ dosing (Figure S13). The broad background peaks were assigned to two components:

- ^13^C Signal from the Activated Carbon; and
- Signal from the probe background.

In regard to the activated carbon, a peak at approximately 120 ppm was observed in all activated carbon samples and was assigned to the aromatic carbon environments, which make up the majority of carbon atoms in an activated carbon material. Peaks at approximately 170 and 70 ppm were observed in some carbons and were assigned to a spinning sideband of the 120 ppm peak. These assignments align with previous results from ^1^H-^13^C cross-polarization measurements of carbon materials.^90–92^ The combined activated carbon signal and probe background signal were accounted for during integration through subtraction of a ^13^C spectrum of the undosed activated carbon from the dosed activated carbon sample, with both spectra recorded using the same relaxation delays and scaled appropriately for sample mass and number of scans.

### Measuring Exchange Rates from QUESP Experiments

QUESP spectra were initially processed with Topspin (phase correction and baseline correction). Further analysis, per the below procedure, was then carried out in Python using the method described below.

#### Calculation of *MTR*_*asym*_

In QUESP experiments, points on the Z-spectrum of the in-pore CO_2_ peak are measured, which track the intensity of the in-pore CO_2_ peak as a function of the center point of the selective presaturation pulse (δ) and of the power of the selective presaturation pulse (ω_1_). The Z-spectrum value for a given presaturation location and power (Z­(δ, ω_1_)) is the ratio of the in-pore CO_2_ peak intensity after saturation (*M*_*sat*_(δ, ω_1_)) compared to the peak intensity in an unsaturated spectrum (*M*_0_), shown in (eq 1).

1 $$Z\left(\delta ,\omega_{1}\right)=M_{\mathrm{sat}}\left(\delta ,\omega_{1}\right)/M_{0}$$

In a QUESP experiment, we set δ to be the difference in chemical shift between the measured peak (in-pore CO_2_) and the labeled peak (in-pore HCO_3_^–^), then record a series of spectra with variable presaturation power (ω_1_) at both δ and – δ.

The magnetization transfer ratio asymmetry (*MTR*_*asym*_) for a given presaturation power (ω_1_) is then calculated empirically (eq 2) by taking the difference in the Z-spectrum of the in-pore CO_2_ peak when we:

- presaturate the opposite (empty) frequency region (*Z*_*ref*_(ω_1_) = *Z*(−δ, ω_1_));
- presaturate the in-pore HCO_3_^–^ region (*Z*_*lab*_(ω_1_) = *Z*(δ, ω_1_)).

2 $$\mathrm{MT}R_{\mathrm{asym}}=Z_{\mathrm{ref}}\left(\omega_{1}\right)-Z_{\mathrm{lab}}\left(\omega_{1}\right)$$

#### Curve-Fitting

To fit the relationship between *MTR*_*asym*_ and ω_1_, we plot *MTR*_*asym*_ against ω_1_^2^ (Figure S12c), and then fit this equation to eq 3:

3 $$y=\frac{x}{x+A\left(x+B\right)}$$

These steps are shown in Figure S12.

If we rearrange eq 3 to $y=\frac{\left(x/\left(x+B\right)\right)}{\left(x/\left(x+B\right)\right)+A}$ , and then set *y* = *MTR*_*asym*_, *x* = ω_1_^2^, *A* = *R*_1*a*_/*f*_*b*_ *k*_*b*_, and *B* = *k*_*b*_(*k*_*b*_ + *R*_2*b*_), this equation becomes eq 4, which is the QUESP equation in the case when *R*_2*b*_ is not negligible.^93^

4 $$\mathrm{MT}R_{\mathrm{asym}}=\frac{f_{b}k_{b}\left(\omega_{1}^{2}/\left(\omega_{1}^{2}+k_{b}\left(k_{b}+R_{2b}\right)\right)\right)}{R_{1a}+f_{b}k_{b}\left(\omega_{1}^{2}/\left(\omega_{1}^{2}+k_{b}\left(k_{b}+R_{2b}\right)\right)\right)}$$

#### Calculating *k*_*b*_ and *k*_*a*_ from Fit

In the case where *k*_*b*_ ≫ *R*_2*b*_, *k*_*b*_ can be immediately obtained as √*B*. Otherwise, √*B* is instead an upper bound for *k*_*b*_. We can therefore calculate a minimum mean lifetime τ_*b,max*_ = 1/√*B.*_R2_b is then measured separately via either a Hahn echo or CPMG experiment. Choice of experiment such that the maximum duration of magnetization evolution (*t*_*max*_) is shorter than τ_*b*,*max*_/100 enables measurement of *R*_2*b*_ on a time scale where minimal exchange occurs, avoiding interference from exchange with other environments with different exchange rates. Obtaining a maximum value for *k*_*b*_ then enables measurement of *R*_2*b*_, since we can choose time scales for measuring *R*_2*b*_ where the exchange process will not influence the measurement. After measuring *R*_2*b*_ independently of *k*_*b*_, the true value of *k*_*b*_ can be calculated using *B* = *k*_*b*_(*k*_*b*_ + *R*_2*b*_). Assuming equilibrium, the reverse rate *k*_*a*_ can then be calculated based on the quantitative NMR-based concentrations (eq 5).

5 $$k_{a}=k_{b}\times \frac{\left[B\right]}{\left[A\right]}=k_{b}\times \frac{\left[{\mathrm{CO}}_{2}\right]}{\left[{\mathrm{HCO}}_{3}^{-}\right]}$$

#### Comparison of Exchange Rates to Literature Measurements of CO_2_ Hydration in Bulk Solution

Our examination of the equilibria involved in CO_2_ hydration and reaction to form HCO_3_^–^ is primarily based on the prior work by Wang et al. (2010),^73^ as well as additional literature.^32,74^ Exchange measurements in this work probe the rates ${\mathrm{CO}}_{2}\overset{k_{b}}{\underset{k_{b}}{⇌}}{\mathrm{HCO}}_{3}^{-}$ . *k*_*a*_ and *k*_*b*_ are pseudo-first-order reaction constants incorporating the concentrations of water, H^+^, and OH^–^ (which are expected to be constant at equilibrium). In bulk, CO_2_ hydration can proceed via a H_2_O or OH^–^-driven route, using the scheme from Wang et al.:^73^

$${\mathrm{CO}}_{2}+H_{2}O\overset{k_{1}}{\underset{k_{-1}}{⇌}}H_{2}{\mathrm{CO}}_{3}$$

$$H_{2}{\mathrm{CO}}_{3}\overset{K_{a2}}{⇌}{\mathrm{HCO}}_{3}^{-}+H^{+}$$

$${\mathrm{CO}}_{2}+{\mathrm{OH}}^{-}\overset{k_{2}}{\underset{k_{-2}}{⇌}}{\mathrm{HCO}}_{3}^{-}$$

*K*_*a*2_ is the acid dissociation constant for H_2_CO_3_; the reaction is assumed to be instantaneous. The reverse reaction, from HCO_3_^–^, is only observed to proceed via formation of H_2_CO_3_, indicating that *k*_–2_ can be disregarded.^73^ From these reaction mechanisms, the pseudo-first-order rates can be related to the relevant concentration of species and the constants *k*_1_, *k*_–1_, *k*_2_, and *K*_*a*2_:

6 $$k_{b}=\frac{k_{-1}}{K_{a2}}\left[H^{+}\right]$$

7 $$k_{a}=k_{1}\left[H_{2}O\right]+k_{2}\left[{\mathrm{OH}}^{-}\right]$$

If *k*_*a*_ and *k*_*b*_ were measured for a bulk solution, then this would enable estimation of the pH based on the exchange rate. However, in the in-pore environment, many of these values are not properly understood, and so, the pH estimation discussed in the main text is a guide.

### Neutron Scattering Experiments

All Small-Angle Neutron Scattering (SANS) measurements in this work were carried out on instrument D22 at the Institut Laue–Langevin (ILL), Grenoble, France, using a neutron wavelength of 5 Å and a beam size of 10 × 10 mm^2^. Electrodes with a diameter of 12 mm and a thickness of 200 μm were punched from the free-standing YP80F and 5 wt % PTFE binder electrode film and mounted in the custom measurement cell described previously.^49^ For wetted samples, 200 μL of either H_2_O or D_2_O was pipetted onto the electrode. The cell was then sealed and allowed to equilibrate for 30 min prior to measurement. Each data set was collected with an exposure time of 30 min and subjected to azimuthal integration and standard transmission and background correction procedures at the instrument. The resulting one-dimensional scattering profiles (Figure 2a) are reported as scattered intensity (arbitrary units) versus the magnitude of the scattering vector, $q=2\pi \times \frac{\sin\theta }{\lambda }$ , where 2θ is the scattering angle and λ the neutron wavelength. The scattering signal prior to the subtraction of incoherent contributions is shown in the inset of Figure S2. The incoherent contribution was approximated by fitting the scattering intensity of 20 data points at 0.9 Å^–1^. For the Kratky representation (Figure 2b), the coherent intensity is multiplied by *q* ^2^.^55^ All raw and integrated data are available at 10.5291/ILL-DATA.1-04-228.

## Supplementary Material



## Data Availability

Experimental data files for the SANS experiments are available at DOI: 10.5291/ILL-DATA.1-04-228. All other data files are available in the Cambridge Research Repository, Apollo, with the DOI identifier: 10.17863/CAM.130157.

## Notes

Additional NMR spectra and data, bicarbonate–carbonate NMR titration data, additional comparisons of NMR-measured uptake with SSA performance, additional information on the QUESP exchange measurement, and literature data for gas sorption of carbon materials (PDF)
