# Supplementary material for: Probing the Phase and Mechanism of Captured CO2 in Supercapacitors by Pore Wetting and NMR Spectroscopy
Source: Chem Mater. 2026 Jun 30;38(13):6566–81. doi: 10.1021/acs.chemmater.6c00749 (PMC13374011; doi:10.1021/acs.chemmater.6c00749)
Supplement: Supplementary file 1 [file cm6c00749_si_001.pdf]

Supplementary Information - Probing the phase and mechanism of captured CO_2 in supercapacitors by pore wetting and NMR spectroscopy

Zeke Coady,[†] Malina Seyffertitz,[†] Benjamin J. Rhodes,[†] Thomas Kress,[†] Amelia Turner,[†] Grace Mapstone,[†] Zhen Xu,[‡] Oskar Paris,[¶] and Alexander C. Forse[*,†]

†*Yusuf Hamied Department of Chemistry, University of Cambridge, Lensfield Road, Cambridge CB2 1EW, United Kingdom*

‡*Department of Materials and Henry Royce Institute, University of Manchester, Oxford Rd, Manchester M13 9PL, United Kingdom*

¶*Chair of Physics, Montanuniversitaet Leoben, Franz Josef-Straße 18, 8700 Leoben, Austria*

E-mail: acf50@cam.ac.uk

Supplementary Figures

(a)

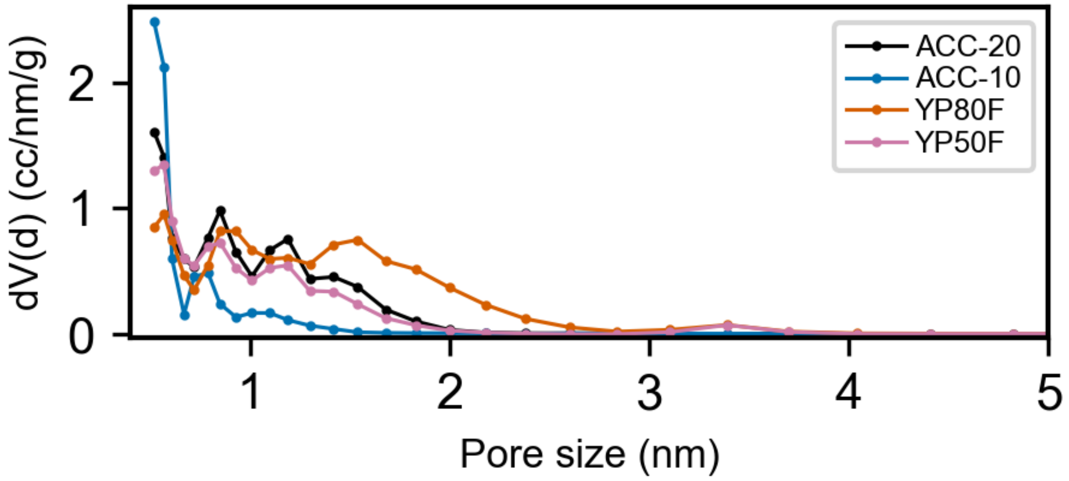

(b)

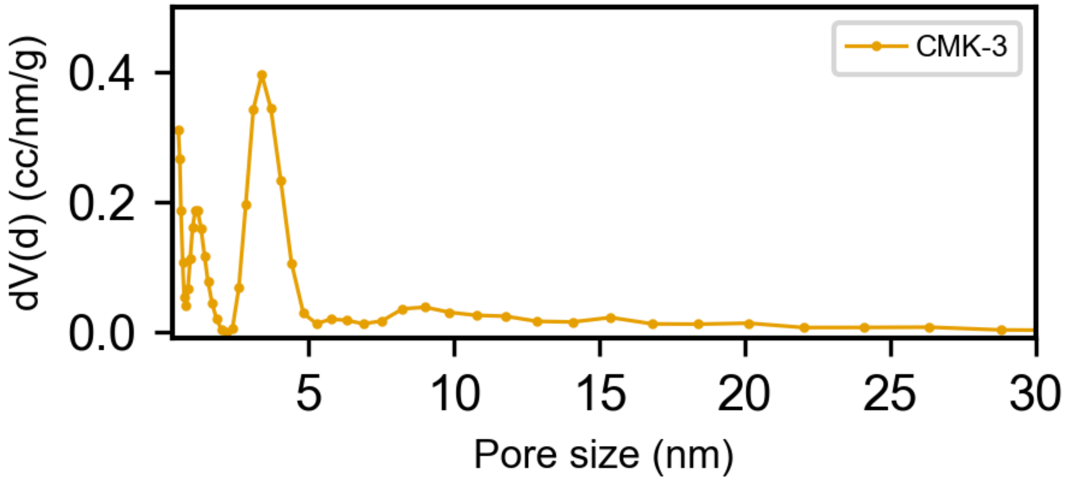

Figure S1: Pore size distributions of the activated carbons in this work, previously reported in Xu et al (2024).[1] Distributions were calculated from N_2 gas sorption isotherms based on quenched solid density functional theory.[2]

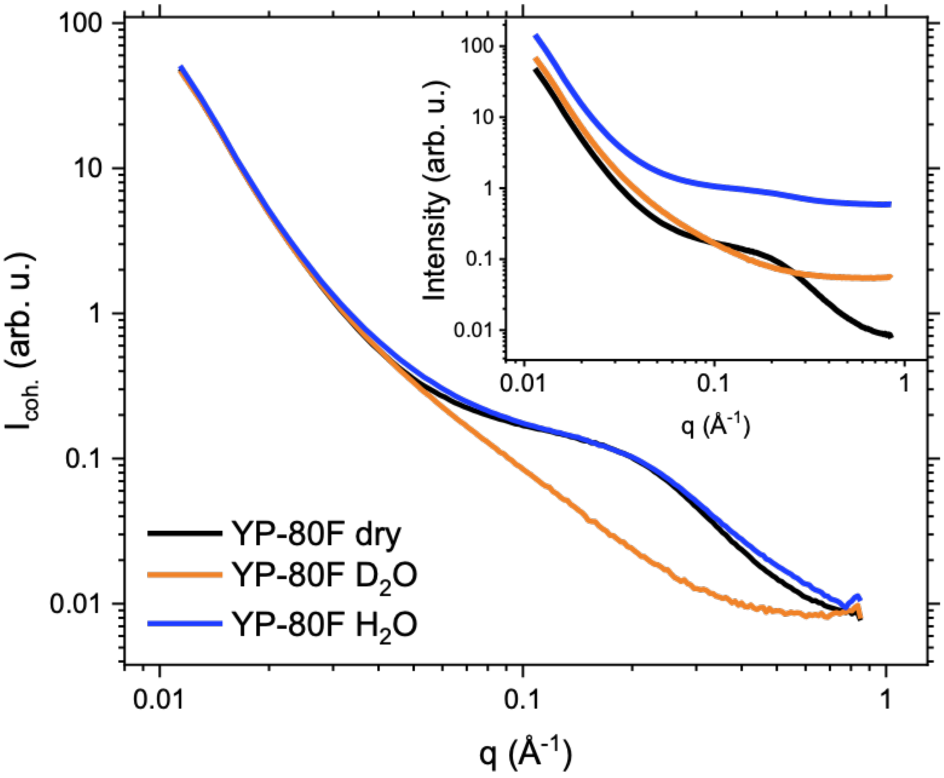

Figure S2: SANS data for dry YP80F (black), and YP80F wetted with D_2O (orange) and H_2O (blue). The main figures shows the coherent neutron scattering signal, while the inset shows the total signal prior to subtraction of the incoherent contribution, approximated by fitting the scattering intensity of 20 datapoints at 0.9 Å^{-1}.

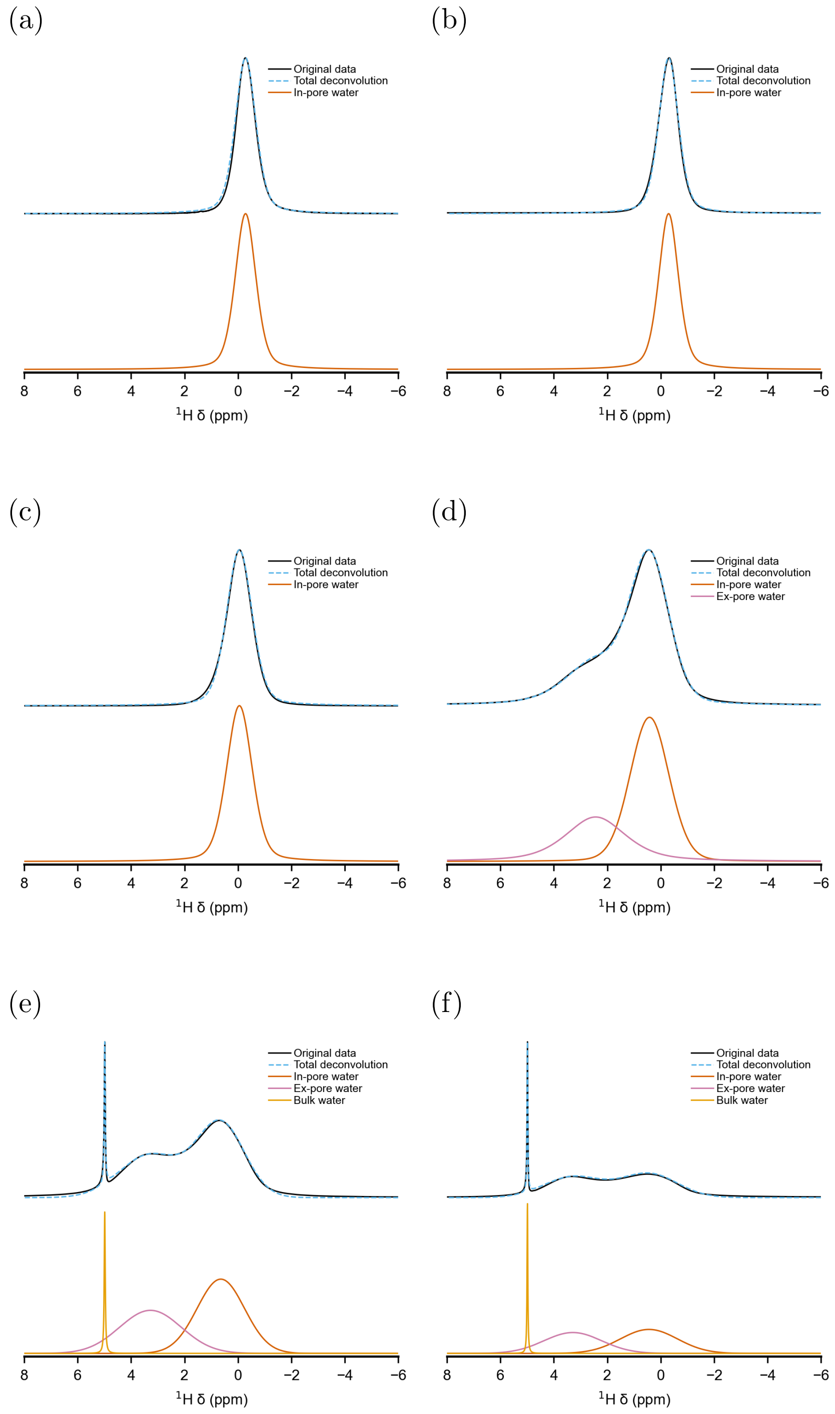

Figure S3: ^{1}H NMR spectroscopy (9.4 T, 5 kHz MAS) measurements and deconvolutions of variably wetted YP80F model electrodes dosed with $^{13}CO_{2(g)}$, showing the three-stage wetting process. Samples at different water:AC mass ratios of a) 0.22, b) 0.52, c) 0.93, d) 1.33, e) 1.54, f) 2.01.

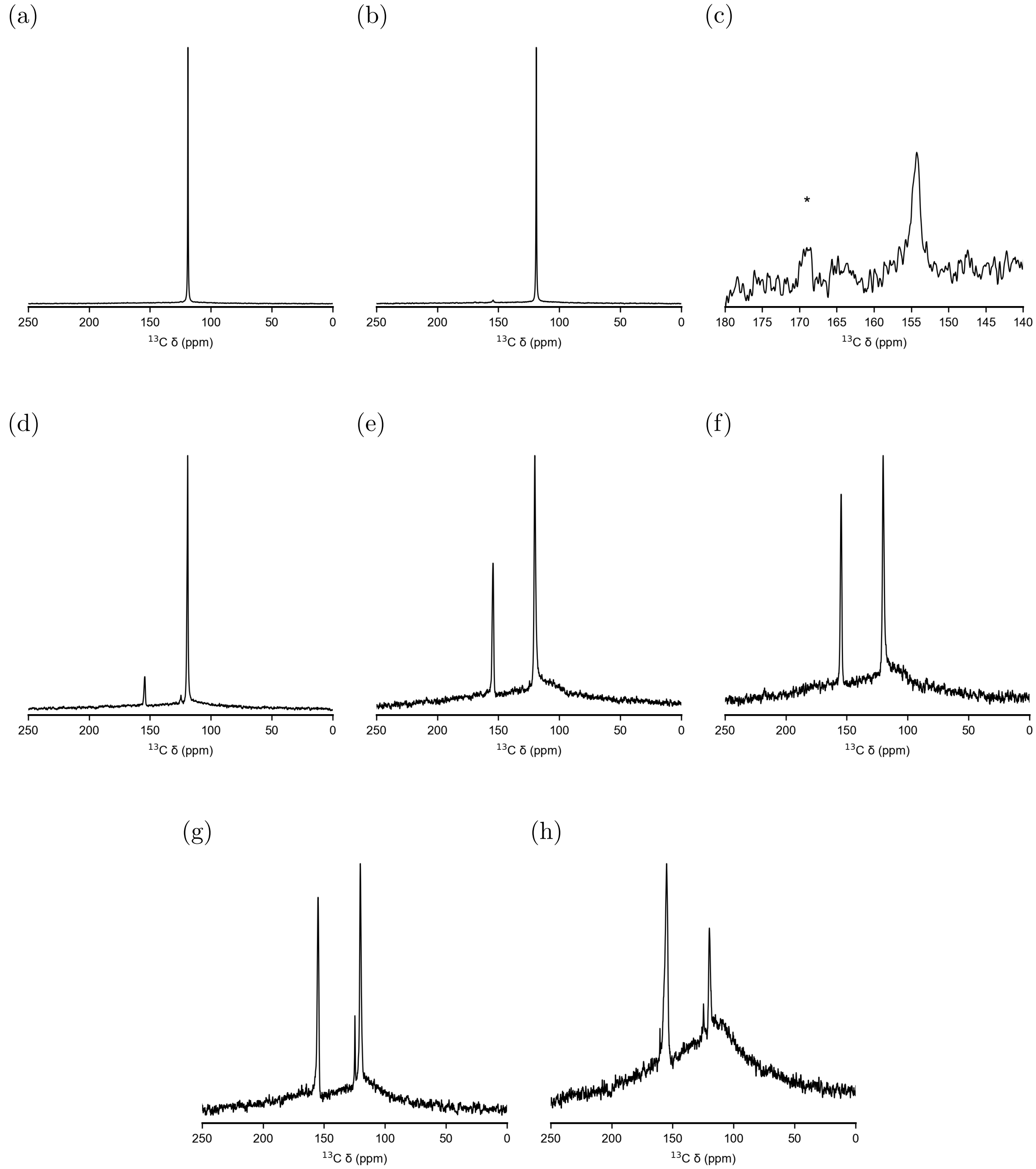

Figure S4: ^{13}C NMR spectroscopy (9.4 T, 5 kHz MAS) measurements of variably wetted YP80F model electrodes dosed with $^{13}CO_{2(g)}$, showing CO_2 and HCO_3^- environments. Samples at different water:AC mass ratios of a) 0.0, b) 0.22, c) 0.22 close-up with asterisk marking a spinning sideband, d) 0.52, e) 0.93, f) 1.33, g) 1.54, h) 2.01.

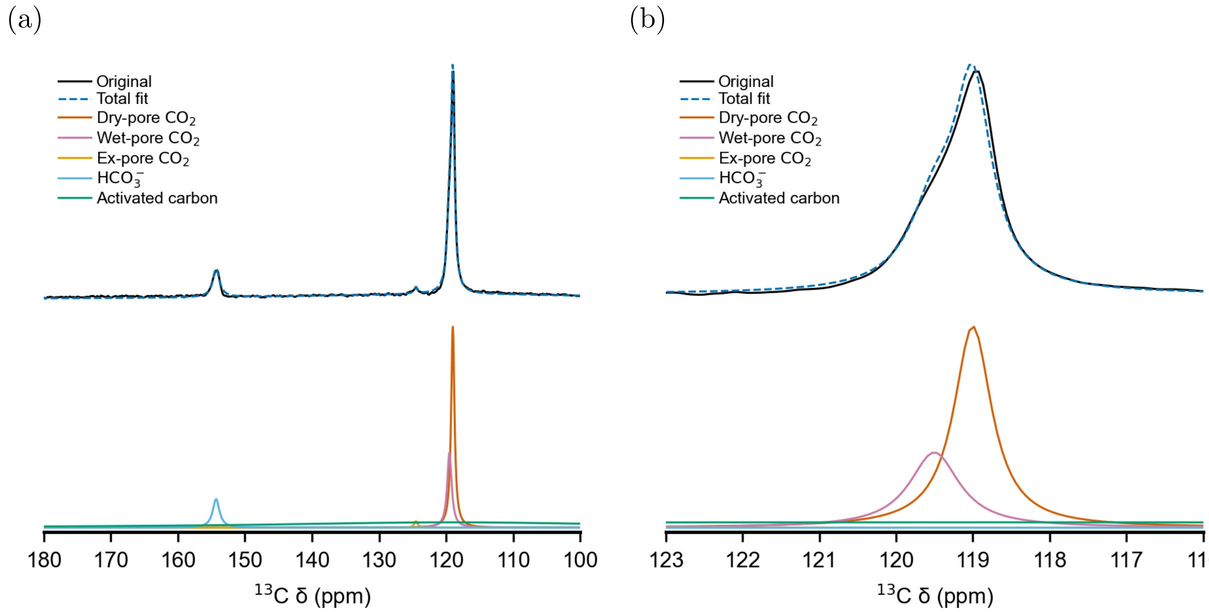

Figure S5: ^{13}C NMR spectrum (9.4 T, 5 kHz MAS) and deconvolution for the 0.52 water:AC mass ratio sample of the variably wetted YP80F model electrodes dosed with $^{13}CO_{2(g)}$, showing a) whole spectrum, and b) close-up on the in-pore CO_2 environments.

(a)

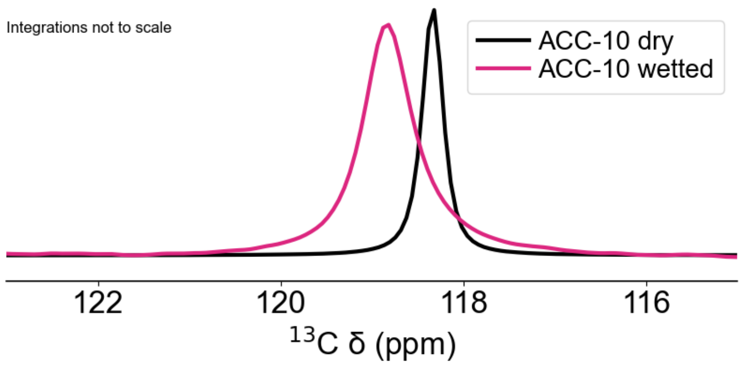

(b)

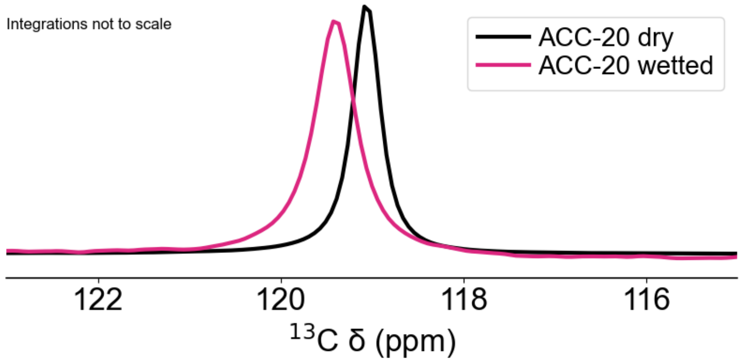

Figure S6: ^{13}C NMR spectroscopy (9.4 T, 5 kHz MAS) measurements of wetted and dry activated carbons dosed with $^{13}CO_{2(g)}$ demonstrate distinct wet-pore and dry-pore environments, with resulting changes in overall uptake for different activated carbons. a) ACC-10, loaded with 1 mL g^{-1} of water prior to $^{13}CO_{2(g)}$ dosing. b) ACC-20, loaded with 1.5 1 mL g^{-1} of water prior to $^{13}CO_{2(g)}$ dosing.

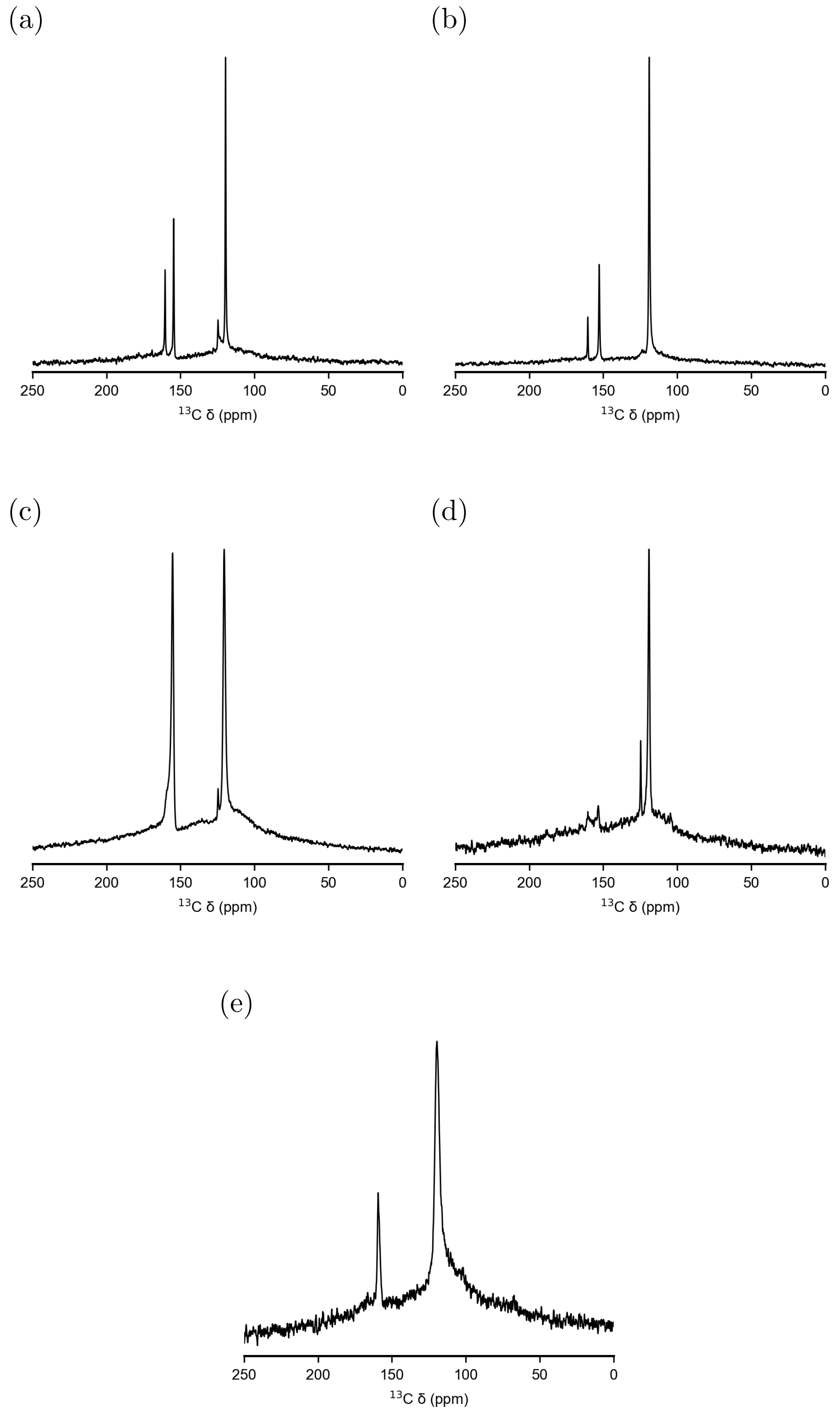

Figure S7: ^{13}C NMR spectroscopy (9.4 T, 5 kHz MAS) measurements of different activated carbon model electrodes soaked with 1 M $Na_2SO_{4(aq)}$ and dosed with $^{13}CO_{2(g)}$, showing CO_2 and HCO_3^- environments. a) ACC-20, b) ACC-10, c) YP80F, d) YP80F, e) CMK-3.

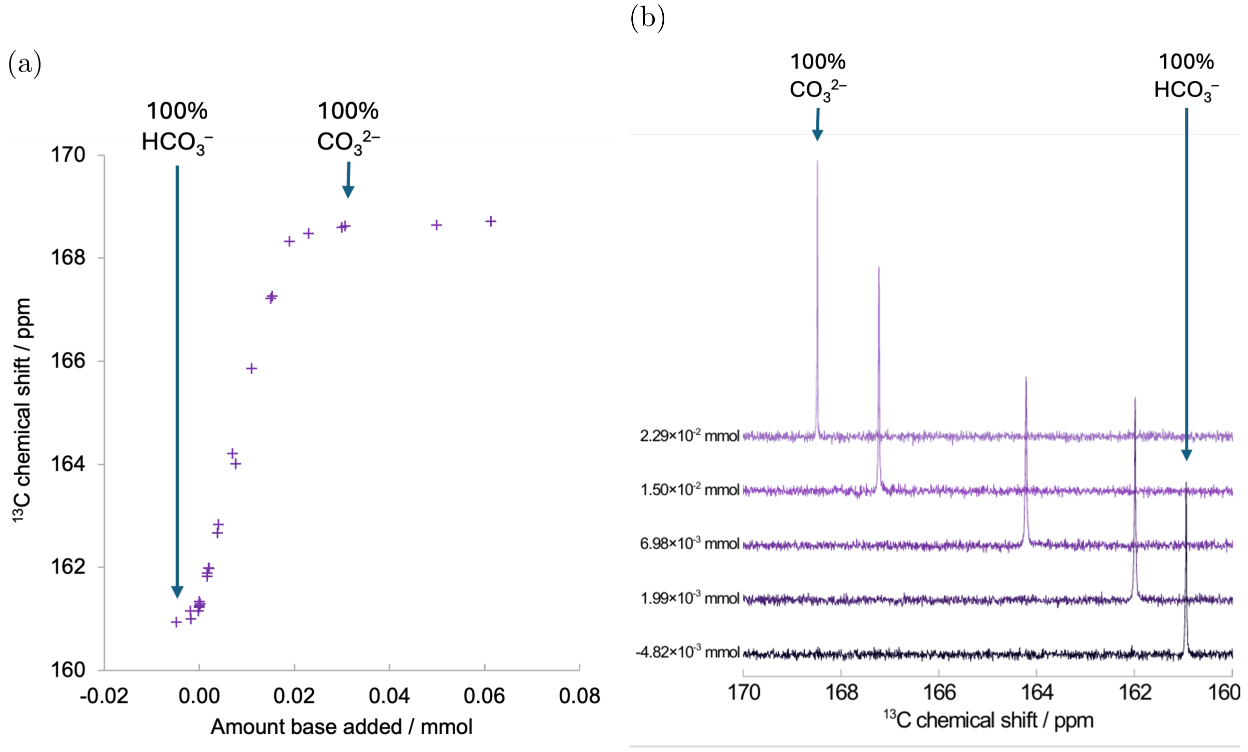

Figure S8: ^{13}C NMR spectroscopy (14.1 T, solution-state probe) calibration curves, prepared by variable addition of NaOH/HCl to a 1 M $NaHCO_{3(aq)}$ demonstrate how the HCO_3^-:CO_3^{2-} equilibrium affects chemical shift in bulk solution. a) Comparison of peak shift to added base/acid (note that addition of acid was denoted as negative addition of base), showing a sigmoidal curve between 161 and 169 ppm. b) Example spectra showing the change in peak shift for selected base/acid concentrations.

(a)

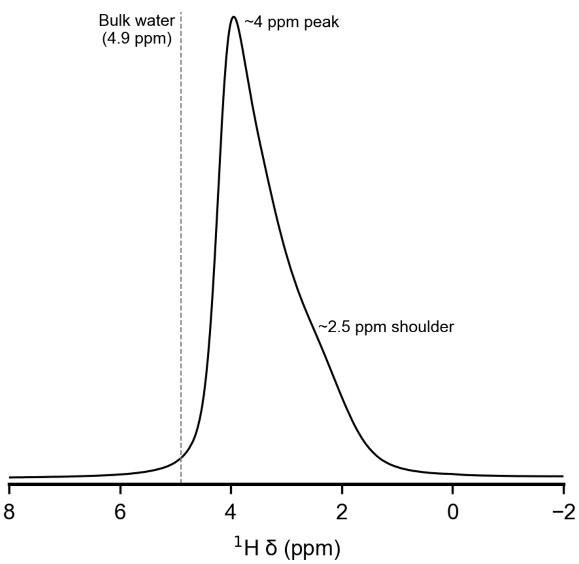

(b)

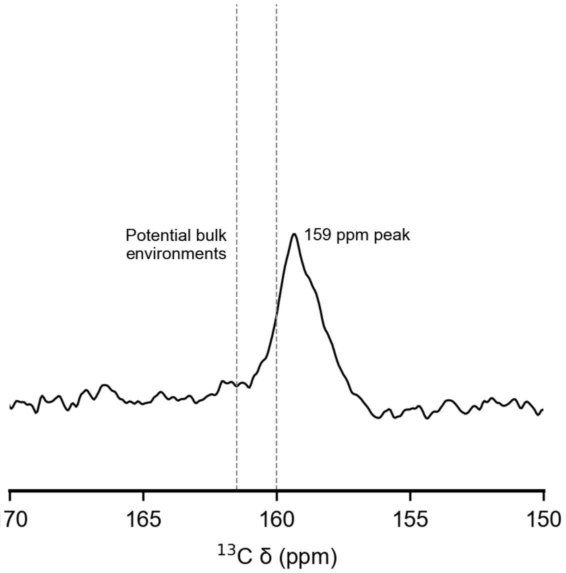

Figure S9: Comparison of ^{1}H and ^{13}C NMR spectra (9.4 T, 5 kHz MAS) for the CMK-3 system suggests the bicarbonate/carbonate environment primarily reflects bicarbonate. a) The ^{1}H spectra shows two overlapping peaks, likely representing in-pore/ex-pore and ex-pore/bulk exchange peaks. The peak separation from bulk water (attributed to the NICS) is observed to be very low (assuming a bulk water chemical shift of 4.9 ppm), being either 0.9 or 2.4 ppm. b) The ^{13}C spectra shows a single bicarbonate/carbonate peak at 159 ppm. Assuming that the peak separation due to the NICS is similar to that observed in water, then the corresponding bulk bicarbonate/carbonate peak shift would be between 161.5 and 160 ppm (shown by the dotted lines), which would indicate this environment only represents bicarbonate.

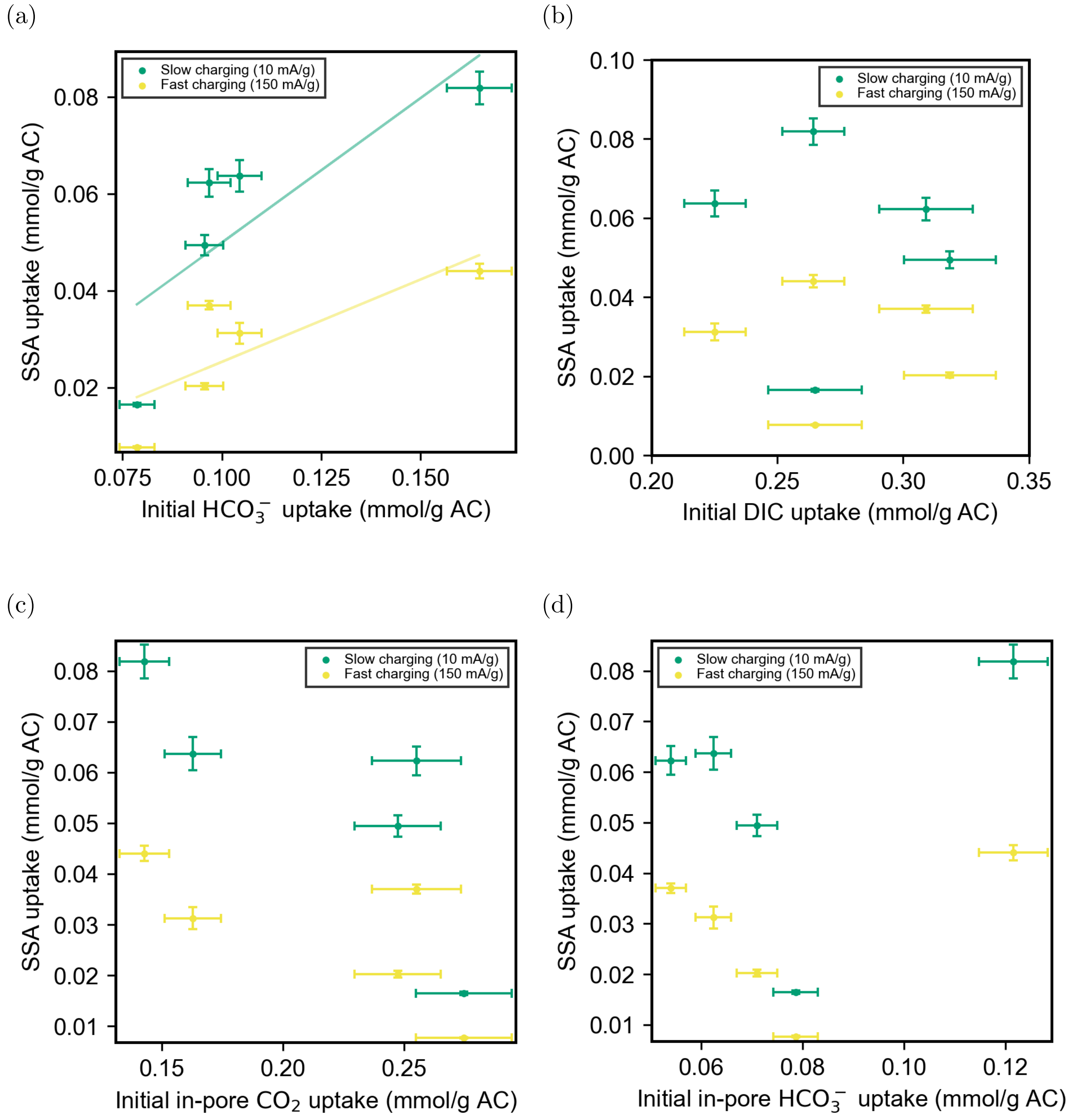

Figure S10: Comparisons of NMR-measured initial uptake of varying CO_2-derived species in model SSA electrodes with the SSA-driven uptake at fast and slow charging rates. For quantification of in-pore species, the HCO_3^- peak in the CMK-3 spectrum was considered to be in-pore. a) Repeat of Fig. 5b). The x-axis shows total HCO_3^-. b) The x-axis shows total in-pore CO_2 and HCO_3^-. c) The x-axis shows in-pore CO_2. d) The x-axis shows in-pore HCO_3^-.

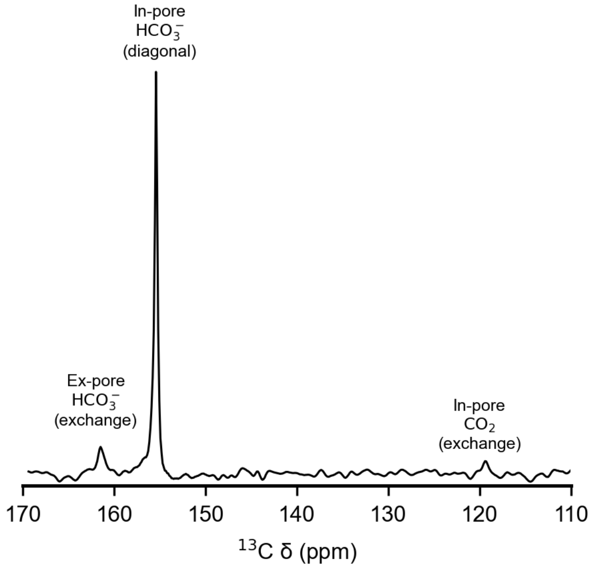

Figure S11: Given a single column slice of the 2D ^{13}C EXSY spectrum of $^{13}CO_2$-dosed ACC-20 + 1 M Na_2SO_4 (Fig. 7) at 155 ppm, examining the in-pore HCO_3^- environment, demonstrates the differing rates of exchange for physical exchange between the pores and chemical exchange between HCO_3^- and CO_2 inside the pores. The large peak at 155 ppm reflects nuclei in the in-pore HCO_3^- environment at the start and end of the mixing time. The small peaks at 160 and 120 ppm reflect nuclei which were in the in-pore HCO_3^- environment at the start of the mixing time, and were in either the ex-pore HCO_3^- or the in-pore CO_2 environment respectively at the end of the mixing time. Note that the in-pore CO_2 peak is only just visible over the noise due to this spectrum being a single slice, and detection of this peak is confirmed by examining the 2D spectrum.

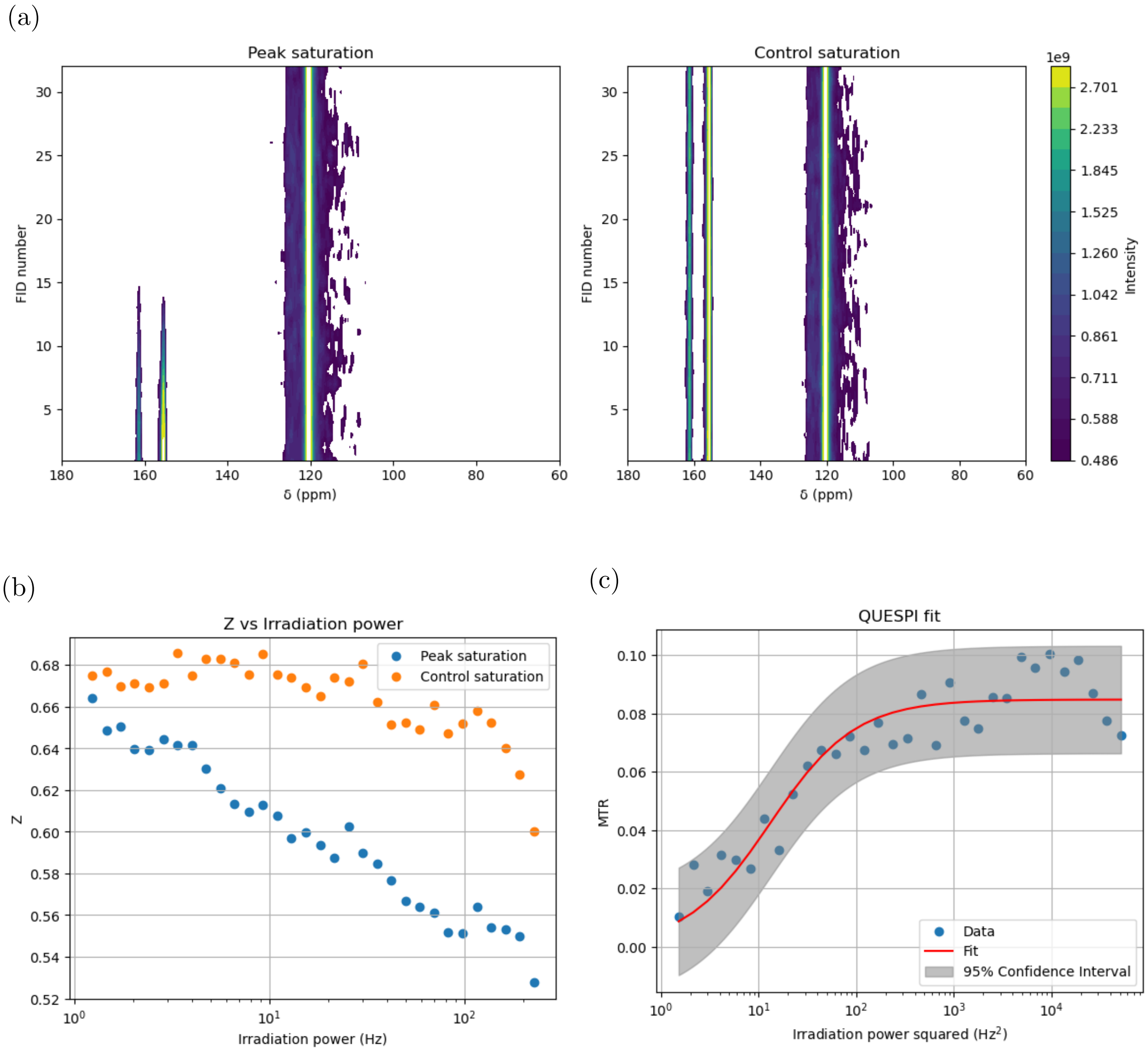

Figure S12: Measurement and fitting of QUESP experiments to a model enables measurement of exchange between the saturated peak (in-pore HCO_3^-) and the measured peak (in-pore CO_2). a) Colormap representation of QUESP experiment shown in Fig. 7b. Saturation power was increased logarithmically with FID number. In the left spectrum, saturation of the in-pore and ex-pore HCO_3^- results in their disappearance. In the right spectrum, saturation of an empty region does not affect the HCO_3^- environment. b) Plot of Z values derived from a) against irradiation power (in Hz), based on **??**. c) Plot of MTR_{asym} values against the square of irradiation power (in Hz), calculated from Z based on **??** and fitted to the model described in **??**.

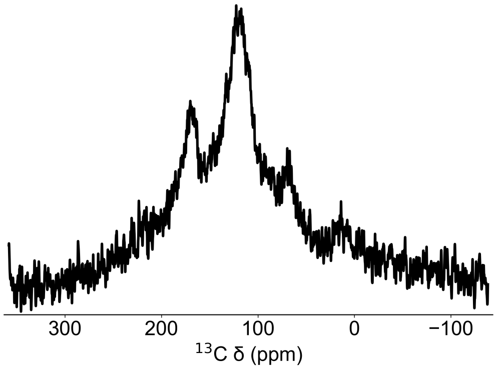

Figure S13: ^{13}C NMR of activated carbon (ACC-20) only, showing the background contribution from the activated carbon (peaks at 120, 170, and 70 ppm) and from the probe background (distortion of baseline).

Supplementary Tables

Table S1: Literature values for BET surface area, accumulative pore volume and average pore size of carbons used in this work, calculated from N_2 sorption isotherms.[1]

Carbon	BET Surface Area (m^2/g)	Accumulative pore volume (cc/g)	Average pore size (Å)
YP80F	2324	1.14	11.3
YP50F	1694	0.73	8.7
ACC-20	2004	0.82	8.9
ACC-10	1094	0.43	5.6
CMK-3	353	1.02	37.1

Table S2: T_1 values measured for each environment in the ^1H spectra for systems shown in Fig. 3a. Values were measured using the inversion recovery pulse sequence. Observation of a 2-component fit in the lowest water:AC ratio is consistent with literature.[3]

System (water:AC ratio)	In-pore T_1 (s)	Ex-pore T_1 (s)	Bulk-like T_1 (s)	Notes
0.22	0.55 / 0.07	-	-	Measured prior to dosing. A 2-component fit is observed for the in-pore environment.
0.52	0.60	-	-	Measured prior to dosing
0.93	0.81	-	-	Measured prior to dosing
1.33	1.0	1.0	-	
1.54	1.2	1.3	1.3	
2.01	1.1	1.1	1.8	

Table S3: Fractionation of water between in-pore, ex-pore, and bulk-like environments based on quantitative ^{1}H NMR spectroscopy. Values are provided as ratios of the mass of water to the mass of electrode (unitless, but described as $g\,g^{-1}$ for clarity). Differences between the system mass (which is based on measurements of the sample during preparation) and the NMR-measured in-pore mass were observed, especially for the 0.22 system, and were ascribed to a combination of skin depth effects and background signal.

System (water:AC ratio)	In-pore water ($g\,g^{-1}$)	Ex-pore water ($g\,g^{-1}$)	Bulk-like water ($g\,g^{-1}$)
0.0	0.00	0.00	0.00
0.22	0.14	0.00	0.00
0.52	0.62	0.00	0.00
0.93	0.95	0.00	0.00
1.33	0.82	0.47	0.00
1.54	0.87	0.66	0.05
2.01	0.99	0.87	0.12

Table S4: T_1 values of the in-pore CO_2 and HCO_3^- peaks in the ^{13}C NMR spectra shown in Fig. 4a). T_1 values were measured using the FLIPS pulse sequence.[4]

System (water:AC ratio)	In-pore CO_2 T_1 (s)	In-pore HCO_3^- T_1 (s)
0.0	0.63	n/a
0.22	0.66	n/a
0.52	0.31	1.0
0.93	0.20	1.2
1.33	0.23	1.1
1.54	0.34	0.71
2.01	0.50	0.50

Table S5: Quantitative NMR spectroscopy-derived concentrations of dissolved inorganic carbon species in different activated carbon electrodes wetted with 1 M $Na_2SO_{4(aq)}$ and dosed with $^{13}CO_{2(g)}$. Electrolyte : AC mass ratios are given in the second column, with sufficient electrolyte introduced to wet each carbon's pores completely; due to the lower pore volume of ACC-10, a smaller volume of electrolyte was used. The observed dissolved inorganic carbon species concentrations reflect a significant solubility enhancement relative to CO_2's solubility in bulk 1 M Na_2SO_4 of 19.6 $\mu mol\,g^{-1}$ at 1 atm of pressure.[5]
*In-pore and ex-pore HCO_3^- could not be resolved separately in the NMR spectrum of CMK-3, so its description as "in-pore" here is arbitrary.

Carbon	Electrolyte:AC mass ratio	In-pore CO_2 ($mmol\,g^{-1}$)	Ex-pore CO_2 ($mmol\,g^{-1}$)	In-pore HCO_3^- ($mmol\,g^{-1}$)	Ex-pore HCO_3^- ($mmol\,g^{-1}$)
ACC-20	1.68	0.163	0.020	0.062	0.042
ACC-10	0.97	0.248	0.007	0.071	0.025
YP80F	1.68	0.143	0.005	0.122	0.043
YP50F	1.73	0.256	0.040	0.054	0.043
CMK-3	1.76	0.274	0.0	0.079*	0*

Table S6: T_1 and T_2 relaxation times for peaks in the ^{13}C NMR spectrum of the $^{13}CO_{2(g)}$-dosed ACC-20/1 M $Na_2SO_{4(aq)}$ system examined in Fig. 7. T_1 values were measured using an inversion recovery experiment. T_2 values were measured using a hahnecho experiment.
*Value could not be measured, indicating likely below 0.010 s.

Variable	In-pore CO_2	Ex-pore CO_2	In-pore HCO_3^-	Ex-pore HCO_3^-
T_1 (s)	0.09	0.03	0.22	0.43
T_2 (s)	0.019	n/a*	0.044	0.010

References

(1) Xu, Z.; Mapstone, G.; Coady, Z.; Wang, M.; Spreng, T. L.; Liu, X.; Molino, D.; Forse, A. C. Enhancing electrochemical carbon dioxide capture with supercapacitors. *Nature Communications* **2024**, *15*, 7851.

(2) Neimark, A. V.; Lin, Y.; Ravikovitch, P. I.; Thommes, M. Quenched solid density functional theory and pore size analysis of micro-mesoporous carbons. *Carbon* **2009**, *47*, 1617–1628.

(3) Song, Y.; Chong, Y.; Raghavan, A.; Xing, Y.; Ling, Y.; Kleinhammes, A.; Wu, Y. Nucleation and Growth Process of Water Adsorption in Micropores of Activated Carbon Revealed by NMR. *The Journal of Physical Chemistry C* **2017**, *121*, 8504–8509.

(4) Wei, R.; Dickson, C. L.; Uhrín, D.; Lloyd-Jones, G. C. Rapid Estimation of T1 for Quantitative NMR. *The Journal of Organic Chemistry* **2021**, *86*, 9023–9029.

(5) He, S.; Morse, J. W. The carbonic acid system and calcite solubility in aqueous Na-K-Ca-Mg-Cl-SO4 solutions from 0 to 90°C. *Geochimica et Cosmochimica Acta* **1993**, *57*, 3533–3554.
